# Deconvolution of High-Dimensional Ion Mobility Data Using Reversible-Jump Markov Chain Monte Carlo

**DOI:** 10.1021/acs.analchem.5c06427

**Published:** 2026-02-26

**Authors:** Jerome Riedel, Marc Safferthal, Gergo Peter Szekeres, Kevin Pagel

**Affiliations:** † 28259 Freie Universität Berlin, Altensteinstraße 23A, Berlin 14195, Germany; ‡ Fritz-Haber-Institut der Max-Planck-Gesellschaft, Faradayweg 4-6, Berlin 14195, Germany

## Abstract

Deconvolution of multicomponent arrival time distributions is known to be a highly challenging task due to the “curse of dimensionality”. Therefore, the development of a robust global optimizer is a crucial milestone in the automated analysis of arrival time distributions for collision cross section extraction and population analysis. Here, we report an approach that combines gas-phase ion transport theory with equi-energy sampling, Bayesian sequential partitioning, and reversible-jump Markov chain Monte Carlo to automatically determine probabilities of deconvolution solutions and predict the number of components in the arrival time distribution. The robustness of the method was evaluated against synthetic and experimental drift tube ion mobility data to find global deconvolution solutions and automatically determine collision cross sections. Analyzing the collision-induced unfolding profile of cytochrome c using the developed pipeline ultimately enables tracking of the conformational ensemble under activation. Additionally, using the same strategy on isomeric *O*-glycan species further revealed the potential for cross-platform collision cross section annotations.

## Graphical abstract

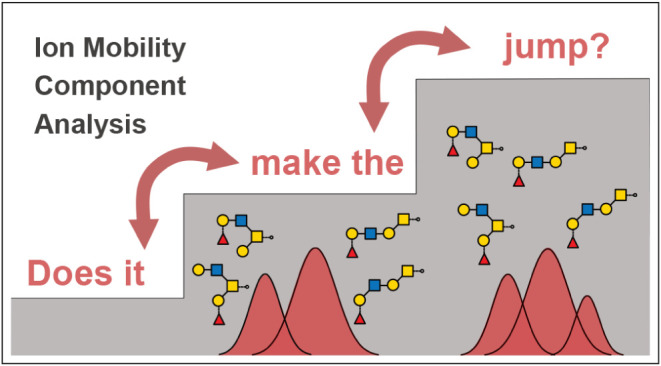

## Introduction

Ion mobility experiments have become a tool of ever-increasing importance in a variety of fields to systematically identify molecules based on their collision cross section (CCS),^1–3^ study their conformational landscape,^4,5^ understand structure–function relationships,^6–8^ and even to differentiate between antibodies based on their collision-induced unfolding (CIU) profile.^9,10^ As such, drift tube ion mobility-mass spectrometry (DTIMS) has repeatedly proven itself as a reliable instrumental technique. DTIMS appeals to researchers for the straightforward instrumental setup, well-defined ion transport characteristics, and low propensity for ion heating. These features enable the rapid and calibration-free measurement of CCSs based on the Mason-Schamp equation, allowing for direct comparison with quantum chemical computations.^11–15^ However, for large and flexible (bio)­molecules, understanding multicomponent arrival time distributions (ATDs)—i.e., multiple conformations or constitutional isomers that contribute to the ATD—remains a challenging task.^16^ In ATDs with a low component count (<3), a deconvolution solution can often be obtained from (regularized) nonlinear optimizers.^17,18^ Most of these optimizers minimize along the gradient of the root-mean-square error (RMSE) and are dependent on the initial starting parameters, algorithm parametrization, and the quality of the parameter bounds for peak amplitude, location, width, and peak-to-peak separation.^19,20^ Therefore, situations arise where the optimizer does not necessarily converge to the global solution but often finds the local minimum closest to the starting conditions.^20^ This limits the large-scale analysis of complex ATDs, since the solutions can be nondeterministic. Additionally, researchers are confronted with the question of how many components contribute to the ATD, also known as the “overfitting problem”.^21^ The complexity of the problem arises from the “curse of dimensionality”^22^ in the solution space of the nonlinear optimization. In the past, it was shown that Markov chain Monte Carlo (MCMC) techniques can be a remedy for complex problems where analytical solutions are not imminent.^23–25^ Through continuous application of a stochastic change, the parameter vector describing the experimental data is updated iteratively until convergence (i.e., a plausible explanation of the data by the parameter vector) is reached.^23,26^ Since the posterior over the parameter space is inferred in MCMC algorithms, probabilistic statements about the quality of model parameters can be made. This is in stark contrast to gradient-based optimizers, where the uncertainty is largely quantified by the fitting error and statistical testing on the residuals.^27^ Although MCMC techniques guarantee a global solution to the problem in infinite time, practically, local convergence can be observed in case of insufficient algorithm parametrization.^28^ To circumvent this, the development of the equi-energy sampler as a special simulated annealing technique provides a pathway to reliably find global solutions.^29,30^ More importantly, within the framework of Bayesian statistics, parameter space constraints can be conveniently expressed in the form of prior probability distributions (priors) that can be directly linked to gas-phase ion transport theory and instrument characteristics.^31^ The equi-energy sampler does not solve the problem of overfitting alone; however, the results can be integrated with a “gold chain” reversible-jump Markov chain Monte Carlo (RJMCMC) approach to extend the inference to the model dimension.^32,33^ Eventually, the probability of explaining the ATD data by *k* components can then be extracted from the posterior over the model dimension targeted by the RJMCMC scheme. Ultimately, this will enable automatic CCS extraction and tracking of conformational ensembles.

In this work, we outline the definition of parameter constraints for DTIMS experiments and their integration in the equi-energy sampler. Global solutions are demonstrated for synthetic ATD data, and the equi-energy results are connected to the RJMCMC algorithm via a density estimation step to automatize predictions about the model dimension. To showcase the relevance of this algorithm, we exploited the complete pipeline on CCS and CIU measurements of cytochrome c 7+ to automatically determine CCS values and track the evolution of the entire conformational ensemble with increasing CIU activation. Besides the protein, we also demonstrated the applicability of this algorithm with isomeric *O*-glycans, which pose a challenging deconvolution problem on DTIMS instruments. In conjunction with the deconvolution procedure, we show that cross-platform comparisons between DTIMS and high-resolution IMS instruments of varying electric field strengths are possible for defined calibration conditions.

## Theory

Evaluation of DTIMS ATD data requires the determination of peak amplitudes, centers, and widths within a linear combination of *k* Gaussian components. Since arrival times of a single species are normally distributed, the model *M*(*t*,Θ) offers a suitable explanation of the observed ATD data *X* according to^34^

1 $$M\left(t,\Theta \right)=\overset{k}{\underset{i=1}{\sum }}\frac{A_{i}}{\sigma_{i}\sqrt{2\pi }}\exp\left(\frac{{\left(t-\mu_{i}\right)}^{2}}{2\sigma_{i}^{2}}\right)$$

where *t* is the arrival time vector of the ATD data and Θ is the parameter vector with entries for the peak amplitude *A*_*i*_ (ion count), center μ_*i*_ (mean arrival time), and variance $\sigma_{i}^{2}$ (ion cloud broadening). To find solutions to eq 1, it is essential to establish adequate bounds on Θ to exclude solutions that do not adhere to ion transport theory in the gas phase. In the context of Bayesian statistics and MCMC techniques, informed parameter bounds can be conveniently expressed in the form of prior probability distributions (priors) that are derived from the ion dynamics in low-field DTIMS.^31^ In the past, multiple physical processes were identified that contribute to the observed peak broadening in DTIMS experiments. Arguably, the three most important contributors are ion injection into the drift tube, diffusion, and Coulomb repulsion.^35^ These three effects not only determine the overall resolution of the experiment, but also impose minimum limits on the explainable ion cloud broadening as a function of ion count and mobility.^35,36^ During the optimization, new values for the number of ions and their mean arrival time will be proposed. The updated ion counts and arrival times can then be used to renew the estimates of diffusion and Coulomb-repulsion-related ion cloud broadening in every iteration. In case of diffusion, ion cloud broadening is driven by temperature and the time *t* ions spend in the drift tube. The ion cloud variance $\sigma_{\mathrm{Diff.},t}^{2}$ due to diffusion is given by^35,37^

2 $$\sigma_{\mathrm{Diff.},t}^{2}=\frac{2k_{B}T}{ze\Delta V}t^{2}$$

where *k*_B_ is the Boltzmann constant, *T* is the temperature, *z* is the charge of the ion, *e* is the elementary charge, and Δ*V* is the effective voltage gradient applied between the respective start and end of the drift tube. For Coulomb repulsion, ion cloud broadening additionally depends on the number of ions with similar mobility that traverse the drift tube as a “macro-ion” with a total charge *Q* = *zeN*_*i*_, where *N*_*i*_ is the number of ions in the *i*-th component and directly proportional to *A*_*i*_. The magnitude of ion dispersion caused by charge interaction is given by^35,38^

3 $$\sigma_{\mathrm{Coul.},t}^{2}=C_{\mathrm{Coul.}}{\left(\frac{3Q}{4\pi \epsilon_{0}\Delta VL}\right)}^{2/3}t^{2}$$

where ϵ_0_ is the vacuum permittivity and *C*_Coul._ is a proportionality factor describing the charge distribution within the macro-ion. Independent of diffusion and Coulomb repulsion, a minimum ion cloud broadening is always present due to ion injection into the drift tube. Here, the degree of ion cloud broadening depends on the shape and duration of the electric field pushing the ions into the drift tube. In case of the commonly found rectangular injection pulses, the ion cloud variance $\sigma_{\mathrm{Inj.},t}^{2}$ after injection can be calculated according to^35^

4 $$\sigma_{\mathrm{Inj.},t}=\frac{t_{\mathrm{Inj.}}^{2}}{12}$$

where *t*_Inj._ is the time duration of the injection pulse. The minimum total variance of the temporal ion cloud dispersion is then given by

5 $$\sigma_{t}^{2}=\sigma_{\mathrm{Inj.},t}^{2}+\sigma_{\mathrm{Coul.},t}^{2}+\sigma_{\mathrm{Diff.},t}^{2}$$

Equation 5 provides an effective infimum, and during the optimization, new parameter updates for $\sigma_{i}^{2}$ cannot fall below. However, deviations larger than the stated infimum are possible due to post-drift-tube dispersion effects and additional ion background (see Supporting Information, Sampling constraints), which are highly instrument- and sample-specific. The updates of the parameter vector then follow a two-step procedure; First, new peak amplitudes and -centers are proposed using a symmetric Metropolis-Hastings (MH) transition kernel. The prior probability pertaining to ion count and arrival time is evaluated to 1/(*b* – *a*), where *a* and *b* are the lower- and upper-limits of the sampling region, respectively (see Figure 1b), and reflect intuitive limits that components cannot be placed outside the region of observed intensity in the ATD and ion counts cannot be negative. Second, new values for the temporal dispersion are sampled as multiples *m* = σ_*t*_/σ_min.,*t*_ of the theory-stated infimum from an exponential probability distribution (see Figure 1c), such that

6 $$\mathrm{Pr}\left(m\right)=\left\{ \begin{array}{ll} 0, & \mathrm{if}\;m<1\\ \lambda \;\exp\left(-\lambda \left(m-1\right)\right), & \mathrm{else.} \end{array}\right.$$

Thus, sampling large deviations from the expected minimum variance decreases exponentially in probability and is disincentivized during the optimization procedure. The degree of deviation that is permitted can be controlled by the parameter λ, where larger values sample σ parameters closer to the stated infimum. It has to be noted that this updating strategy breaks detailed balance due to jointly updating two parameters in a single step; however, it has been shown that convergence can be enhanced as long as local balance (i.e., the same probability for the local forward and backward move) is observed.^39,40^ This is guaranteed by sampling changes in arrival time and ion count from a symmetric normal distribution, which directly determines the sampling of *m*. After definition of the parameter constraints, calculation of the best parameter vector for describing the ATD under the model constraints follows a three-step algorithm. At the beginning, the fixed-dimensional posterior Pr­(Θ_*k*_|*k*, *X*) for *k* components is inferred using the equi-energy sampler (see Supporting Information, Equi-Energy sampling).^24,29^ Markov chains at different temperatures are successively started with mixing of equi-energy parameter vectors enabled, so that higher-energy states can propagate into lower-temperature chains. This ensures crossing of energy barriers at lower temperature (see Figure 2a). At the lowest temperature, samples are directly drawn from the posterior describing the ATD. The equi-energy sampling process is repeated for all number of components *k* that are suspected to yield a valid description of the ATD data. Then, each inferred posterior for a target dimension *k* is subjected to the Bayesian sequential partitioning (BSP) algorithm (see Supporting Information, Bayesian sequential partitioning) to convert the posterior samples of the equi-energy step into a discretized posterior (see Figure 2b).^41,42^ Finally, all discretized posteriors are used in the reversible-jump Markov chain Monte Carlo (RJMCMC) stage to sample jumps that increase or decrease the number of components to explain the ATD (see Supporting Information, Reversible-Jump Markov chain Monte Carlo).^32,33,43^ The posterior Pr­(*k*,Θ_*k*_|*X*) that is inferred by the RJMCMC algorithm provides the probability for the number of components under the ATD as well as the probability of observing a certain parameter combination in the parameter vector Θ_*k*_ via the dimension and parameter combination that was proposed and accepted most often during the optimization (see Figure 2c).

**Figure 1:**
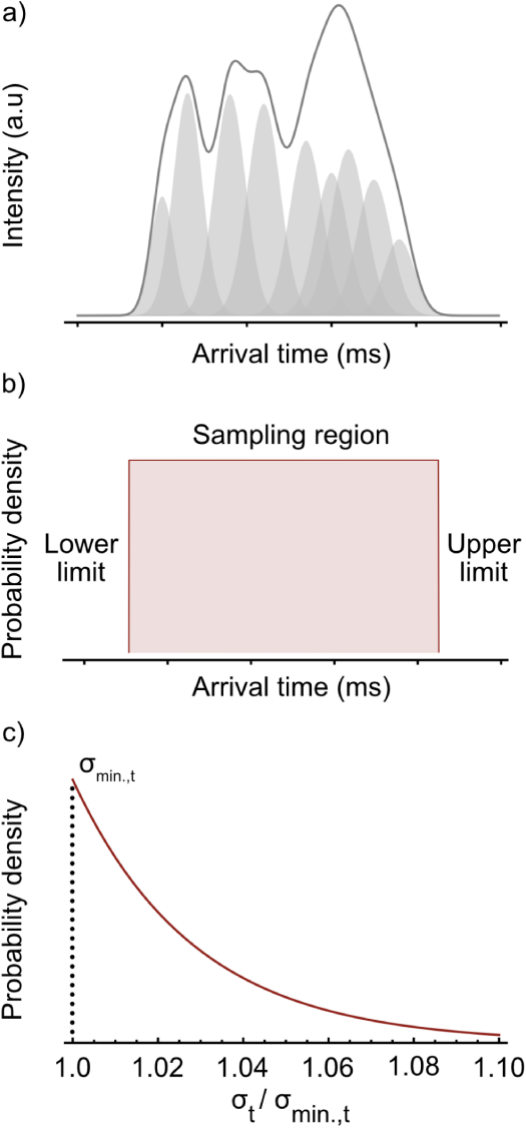
Sampling constraints for ATD deconvolution. (a) Example of a synthetic, multicomponent ATD. Sampling regions for arrival time and ion count can be directly inferred from the width and height of the ATD. (b) Uniform prior as used for the sampling of arrival times. Arrival times can only be sampled in the nonzero intensity regions of the ATD. (c) Exponential distribution (λ = 35) that was used to propose new values for ion cloud broadening.

**Figure 2:**
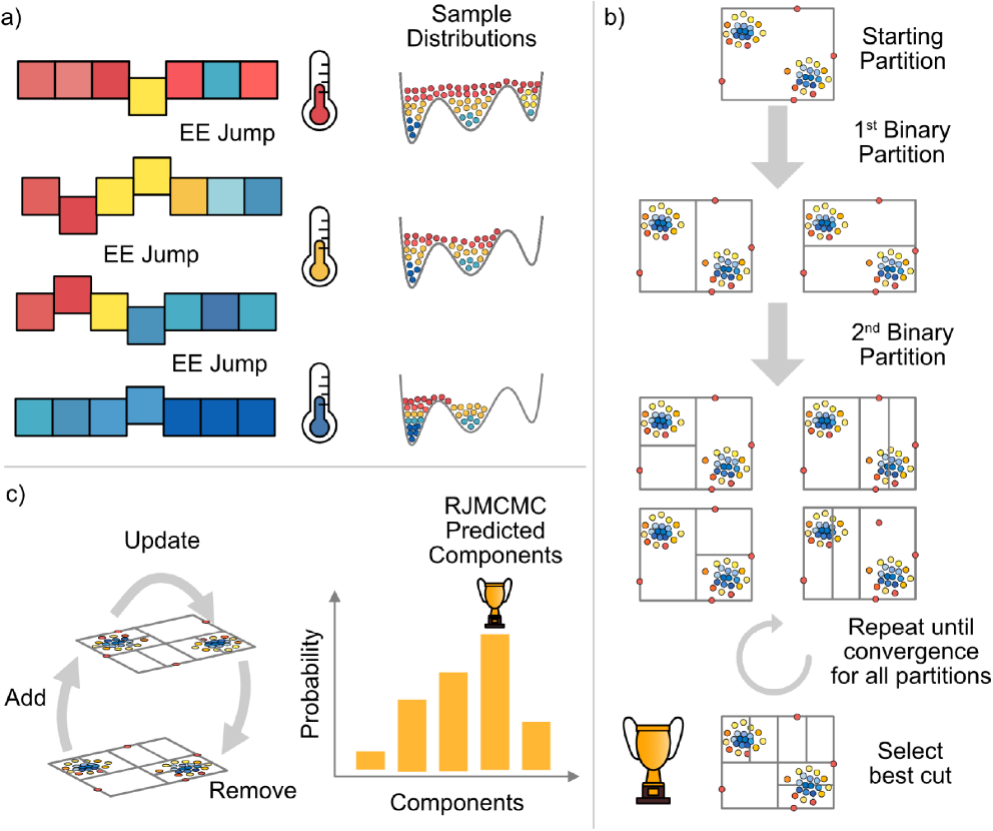
RJMCMC pipeline to automatically select the number of components. (a) Equi-Energy (EE) sampling at different temperatures for a fixed dimension. At high temperatures, the complete sample space is accessible. (b) BSP of the sample space after equi-energy sampling. Recursive partitioning of subregions yields density estimates by counting the number of samples in each subregion. (c) Dimension inference in the RJMCMC scheme by sampling jumps from subregions of high density. The predicted number of components can be inferred as the mean over the dimension posterior.

## Computational Details

Validation of the RJMCMC pipeline was first performed on synthetic ATD data, which were calculated according to eqs 2–4) (see Supporting Information, Algorithm validation). Simulated experimental parameters were set to the same parametrization of the modified Waters Synapt G2-S, which was later used for all IMS measurements (see Supporting Information, Instrument parameters). Ion characteristics for validation were chosen to resemble the deconvolution problem of proteins with high charge states and multiple conformations. Model inference on experimental data was started with an estimation of the lowest dimensional model that provides a reasonable description with *k* components and was extended to the interval [*k*, *k* + 3]. The parametrization of the equi-energy sampler and Bayesian Sequential Partitioning adapt optimized parameters that were previously stated (see Supporting Information, Algorithm parametrization).^29,41,42^ A detailed description of the deconvolution procedure is provided in the Supporting Information.

## Experimental Details

All samples were measured in a modified Waters Synapt G2-S with nanoelectrospray ionization. The initial IMS cell was replaced with a constant field drift tube (*L* = 0.2505 m) that has been described in detail elsewhere.^44^ In the CIU experiments, the collision voltage was scanned between 0 and 30 V in steps of 3 V. No additional quadrupole selection was performed. Cytochrome c from equine heart (Sigma-Aldrich, SDS-PAGE ≥ 95%, 10 μM, U.S.A.) was measured in 200 mM ammonium acetate solution. *O*-glycan samples from porcine gastric mucin (Sigma-Aldrich, USA) were prepared as described previously.^45^ The released *O*-glycans were dissolved in 50 mM ammonium acetate solution and ionized by electrospray ionization.

## Results and Discussion

### CCS Deconvolution

The measurement of CCSs involves a series of drift-voltage-dependent ATD measurements (stepped-field method), where different voltage gradients are applied across the drift tube. The change in arrival time as a function of the inverse drift voltage follows a linear relation, where the slope is proportional to the ion mobility *K*. Ion mobility measurements offer an ideal testing ground for the RJMCMC approach. Incorrect deconvolution solutions are identified by a strong nonlinear character in the ion mobility extraction step. Furthermore, it is essential for the predicted number of components in each ATD to remain constant across all drift voltages. To that end, the cytochrome c 7+ (*m*/*z* = 1766) charge state species was analyzed at six different drift voltages. For the deconvolution of large and flexible (bio)­molecules the equi-energy sampler resolves different conformational families of similar CCS present in the ATD, henceforward referred to as a (conformational) component. Each component consists of multiple conformers that cannot be reduced to a single representative structure but are a qualitative and instrument-specific average of the family. In the equi-energy stage, models with 5–11 components were tested. Dimensions 5–7 are immediately ruled out due to a failure to provide low RMSE deconvolutions within the parametrization of the model. For the remaining dimensions 8–11, the results of equi-energy sampling were subjected to BSP and eventually RJMCMC. Based on the average predicted dimension over all voltage dependent ATD measurements, the predicted number of components is 9.6 ± 0.6 after RJMCMC (see Figure 3b). As the predicted value falls between two integers, the CCS extraction was performed for 9 and 10 components. Here, both dimensions show a linear trend, however, in terms of *R* ^2^, a better description was given by the 10-component model (see Figure 3c). This is consistent with the RJMCMC results for the synthetic data, where the difference between a higher and lower dimension is often given by the merger of two neighboring components (see Figures S4 and S5). This merged component then behaves comparably normal in the deconvolution across drift voltages; however, it also provides a worse description of the ATD data. A summary of the corresponding cytochrome c CCSs is given in Table 1. For the CCSs of all 10 predicted components, a mean error of 1.8% is calculated. The predicted components cover a CCS range from 1280 to 2000 Å^2^ and are in good agreement with literature values that were previously reported from drift tube-, traveling wave- (TWIMS), and trapped ion mobility (TIMS) measurements.^46,47^ As shown by the population analysis of the synthetic 9-component deconvolution (see Table S4 and Figure S2) statements about absolute population values can be ambiguous for >7 components. This is further observed in the fact that across all ATDs, the relative and absolute heights of individual components change (see Figure 3a and Figure S10). This behavior is likely linked to the operational setup of the Waters Synapt G2-S instrument as well, where 200 TOF injection pulses limit the overall number of available data points in the mobility dimension. Hence, individual components only have support of 5–10 data points in the observed drift range, which limits the overall quality of the deconvolution solution.

**Table 1:** Helium Drift Tube CCSs for Each Predicted Component by RJMCMC in the Arrival Time Distribution of Cytochrome C 7+^a^

Component	${\mathrm{CCS}}_{\mathrm{DT}}^{\mathrm{He}}$	Error	*R* ^2^
1	1284	25 (2.0%)	0.998
2	1349	25 (1.9%)	0.999
3	1395	25 (1.8%)	0.999
4	1479	24 (1.6%)	0.999
5	1558	41 (2.6%)	0.997
6	1637	25 (1.5%)	0.999
7	1709	19 (1.1%)	0.999
8	1827	14 (0.8%)	0.999
9	1905	42 (2.2%)	0.998
10	1962	43 (2.2%)	0.998

a The average CCS error is 1.8%. All values are given in Å^2^.

**Figure 3:**
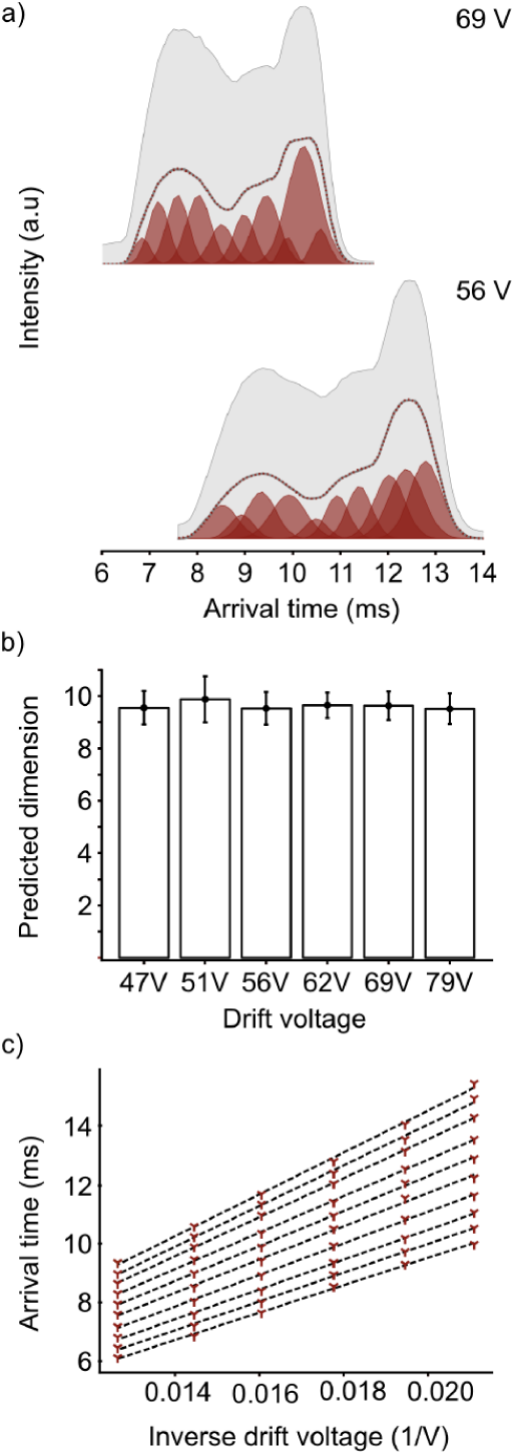
CCS analysis of cytochrome c 7+. (a) ATDs at different drift voltages. Ion background from cytochrome c 6+ and 8+ and adducts is shown in gray (see Figure S9). (b) Average predicted dimension after RJMCMC across all drift voltages. The average number of components is 9.6. (c) Linear fit of the arrival times against the inverse drift voltage for the predicted 10-components. High linearity was observed across all component arrival times.

### CIU Deconvolution

CIU experiments pose an interesting challenge for large-scale data analysis. Here, an automated deconvolution workflow that is sensitive to the ion background can help to entangle the CIU results of multiple ions within the same CIU run in parallel and facilitate fast data evaluation.^48^ Cytochrome c has been extensively studied with CIU to understand unfolding/refolding dynamics, stability regimes, and activation barriers.^46,49,50^ In the past, it has been shown that under collisional activation, the initial conformer ensemble of cytochrome c transitions from a very broad set of folded and unfolded states to mostly unfolded geometries.^51^ Early studies suggested the presence of at least five different conformer families A–E that could be converted upon activation.^52,53^ Recent measurements revealed at least 8 different conformer families of cytochrome c 7+ and conversion dynamics upon activation were shown to take place in the ms-regime.^54,55^ The measurement of the CIU profile of cytochrome c 7+ is shown in Figure 4a, along with the predicted transition pathway from the RJMCMC deconvolution for a supervised set of model dimensions in Figure 4b and S11. The results after RJMCMC indicate that cytochrome c 7+ undergoes three distinct phases. From 0 to 6 V, the predicted number of components in the ATD stays constant (9 components). Compared to the ATD at the identical drift voltage without activation, funneling ions through the buffer gas-filled collision trap at 0 V leads to a different ATD profile (see Figures S10 and S11). This change in the ATD potentially provides an explanation for the difference in calculated component count as a result of minor activation in the trap. At early collision voltages, the predicted peak centers of each component do not shift, indicating that the conformational ensemble is stable at low activation energies. At 9 V, a minor decrease in the number of components (1 component) was observed as the most compact geometry disappeared. Furthermore, the predicted centers of some components shift. Possibly, the shifted peak centers indicate the presence of metastable geometries in the transition between two stable components deconvolved at 6 V.^51,52^ For the range of 0–9 V, the ATD is overall dominated by stable, more compact geometries, which is in good agreement with earlier observations.^52,54^ Between 12 and 15 V a stark decrease in the number of components is observed. Here, the predicted number of components decreases to 5 and large shifts in the arrival time are observed. This indicates that a significant activation barrier is crossed, which finally allows the transition for the majority of compact geometries into extended structures. In the following range of 18–30 V, the predicted number of components remains constant. This reproduces previous observation that a continued unfolding of cytochrome c only takes places at much higher collision energies, which were not reached during the experiment.^51^ Compared to the initial ATD at low collision voltages, the complete high-activation ATD is dominated by less compact structures that do not undergo further structural changes. The previous bimodal distribution is replaced by a unimodal distribution around conformer families of the extended type for which the predicted arrival times remain constant.

**Figure 4:**
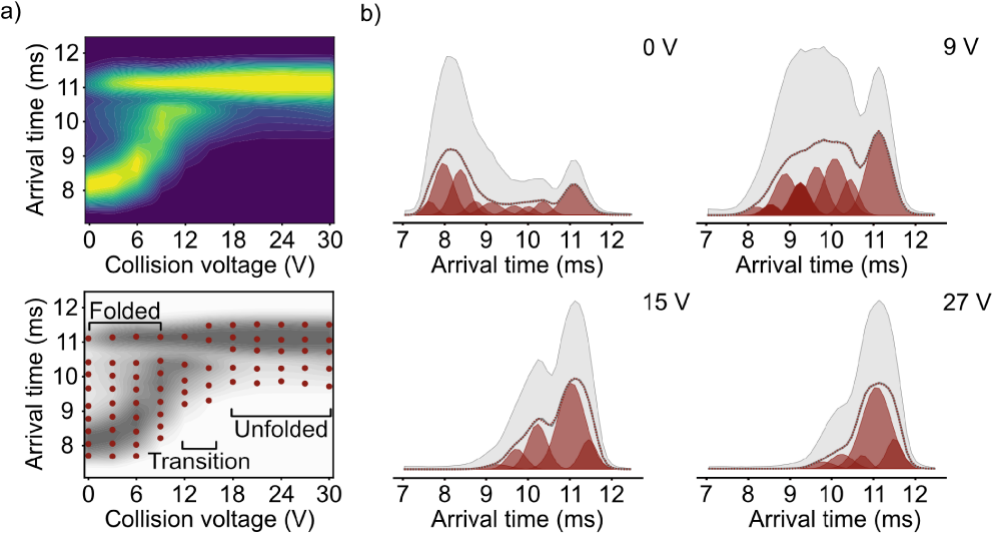
Deconvolution of the cytochrome c 7+ CIU profile. (a) ATDs of cytochrome c 7+ at different collision voltages between 0 and 30 V with predicted arrival times (red) after equi-energy sampling for the dominant dimension (bottom). (b) Deconvolution solutions at selected drift voltages for the dominant dimension.

### *O*-Glycan Deconvolution

Since the resolution of nonuniform electric field IMS instruments is usually higher, it is important that the number of resolved components on the higher-resolution instrument is not immediately transferred as an initial guess for the number of components the deconvolution must find on the DTIMS instrument. Instead, it is crucial that any deconvolution strategy infers the appropriate number of components that can be maximally resolved automatically. One such analytical application is the observation of *O*-glycan constitutional isomers in porcine gastric mucin (PGM) that are readily resolved with LC-TWIMS or TIMS (see Figure 5) but require calibration against a dextran ladder to calculate their CCS.^45^ The applicability of the dextran ladder for calibration has been previously demonstrated for *N*-glycans;^56^ however, a formal validation for *O*-glycans remains to be provided as only very limited DTIMS data are available. Previously measured CCSs for TIMS are summarized in Table 2 and are generally highly reproducible within ≈2% between both instruments. For a comparison with DTIMS, identically prepared *O*-glycan samples from PGM were analyzed on the previously described DTIMS instrument in nitrogen buffer gas. ATDs for the ions with *m*/*z* 749, 895, and 952 are extracted (see Figure 5) and deconvoluted under consideration of the ion background for models with 2–4 components using RJMCMC, where the upper limit is inferred from the higher-resolution TIMS data. For the DTIMS instrument used in this work, the deconvolution of *O*-glycans with *m*/*z* 895 and 952 poses an interesting challenge, since the CCS difference of 6–7 Å^2^, measured on TIMS is close to the resolution limit of the specified priors. The deconvolution of isomers with a CCS difference of ≈2 Å^2^ should not be possible for the implemented constraints and therefore must not be reported by the software as an appropriate solution. Hence, in case of the isomers with *m*/*z* 749, it is expected that the number of components is at least one lower than immediately visible in the TIMS data, because isomers with CCS_TIMS_ of 273 and 275 Å^2^, respectively, will appear merged.

**Figure 5:**
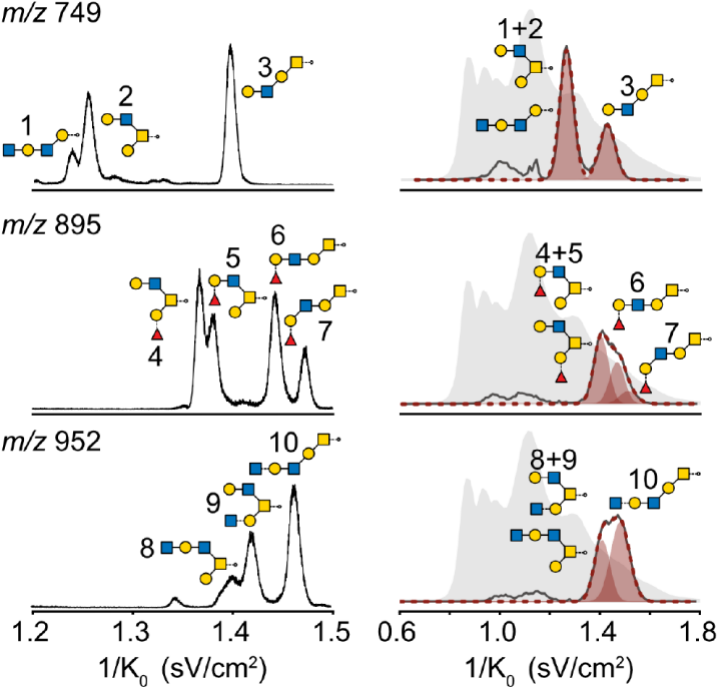
(Left) Reference measurement on a Bruker timsTOF Pro. Peaks are annotated with the *O*-glycan structures in SNFG nomenclature according to.^45^ (Right) Deconvolution solutions of the dominant dimension after RJMCMC at 138 V drift voltage converted to the mobility dimension. The ion background of the sample is shown in gray and the extracted ion current in black. Deconvolution solutions were scaled and are shown in red.

**Table 2:** Comparison of PGM *O*-Glycan CCSs Measured on a Modified Synapt G2-S DTIMS and Bruker timsTOF Instrument^a^ ^45^

*m*/*z*	Isomer	${\mathrm{CCS}}_{\mathrm{TIMS}}^{N_{2}}$	${\mathrm{CCS}}_{\mathrm{DT}}^{N_{2}}$
749	1	245	251 ± 1
	2	247	
	3	281	285 ± 2
895	4	273	283 ± 2
	5	275	
	6	290	296 ± 2
	7	297	308 ± 5
952	8	280	276 ± 4
	9	286	
	10	295	291 ± 4

a Error estimates are given for the 3σ confidence interval. All values are in Å^2^.

Starting with the deconvolution of the *m*/*z* 749 isomers, a full description of the DTIMS ATD is only given by three components, but equivalent CCS values are reported for the model with two components for the main peaks. Here, the third component is consistently located between the two main peaks and is probably attributed to the presence of ion background within the integration window of the *m*/*z* 749 species. A similar ion background can also be observed in the TIMS mobility distribution and will appear compressed between the two main peaks in the DTIMS data. As a result, the third component was omitted. The deconvolution of *m*/*z* 895 and 952 provides reasonable descriptions of the ATD for models with two and three components that both exhibit strong linearity across all drift voltages (see Figures S12–S14). Estimating the dimensionality of the model using RJMCMC without a prior that is discouraging higher dimensions (i.e., simply based on the error of the model) yields an average dimension of 2.6 and 2.2 for *m*/*z* 895 and 952, respectively, indicating that for *m*/*z* 895 the ATD provides enough information for an additional third component. Inspection of the corresponding ATDs, especially at the highest drift voltage and resolution, indicates the two-component model fails to accurately capture intensities at the tail end of the distribution (see Figure 5, isomer 7). Additionally, the relative intensities are qualitatively similar to the intensities observed in TIMS. In case of *m*/*z* 952, the resolution of the drift tube instrument and deconvolution is not sufficient to explain a third component. Moreover, the corresponding ATD measured on the TIMS device indicates a harder separation problem in terms of the 1/*K*_0_ difference between the two peaks at the left tail end of the distribution, rendering an identification of this component impossible on a DTIMS device. Since the expected CCS difference is close to the resolution limit of the deconvolution, generally higher uncertainty in the calculated CCS is found for isomers with *m*/*z* 895 and 952. The results from the TIMS and DTIMS comparison show good agreement between platforms with a mean absolute error of ≈3% (components close in CCS were weighted according to their intensity) and reproduce previously reported deviations for dextran/*N*-glycan DTIMS and TWIMS comparisons.^56^ On average, a tendency for an upward deviation between TIMS and DTIMS CCSs was observed, but cross-validation with the deconvolution results supports the applicability of the dextran ladder as a calibrant for *O*-glycans in the analyzed mass range. Further deviations might also be explained by additional low-intensity ions of similar intensity that possibly further alter the inferred arrival times. Thus, cross-platform comparison of ATDs after deconvolution may have the powerful potential to support CCS values measured by calibration for complex separation problems.

## Summary and Outlook

In this work, we outlined the combination of equi-energy sampling with a Bayesian model for ion transport theory to derive global deconvolution solutions for arrival time distributions (ATDs) of all molecular ions that are described within the low-field limit of drift tube ion mobility instruments. Successive application of Bayesian sequential partitioning (BSP) and reversible-jump Markov chain Monte Carlo (RJMCMC) permits the automated extraction of component count based on a supervised set of model dimensions probed in the equi-energy stage. Due to the strong Bayesian character of the complete algorithm, the confidence in the deconvolution solutions and dimension determination can be made quantifiable at every stage. The performance evaluation of the outlined approach on experimental ion mobility data of cytochrome c and *O*-glycans has demonstrated the ability to automatically predict the number of components in ion mobility measurements and, based on that, calculate collision cross sections (CCSs) of every predicted component. In the case of collision-induced unfolding (CIU) experiments of cytochrome c, tracking the conformational ensemble with RJMCMC across applied collision voltages has confirmed previous observations of conformer population stability, activation, and dynamics. Thus, calculated predictions can also be integrated with *in silico* conformational sampling tools and molecular simulations to compare extracted components to theory for inference of atomic-level geometries and transition energies. In the future, the equi-energy sampler can be adapted to incorporate ion broadening terms for other IMS instruments with different underlying ion transport physics. This requires a set of newly derived analytical equations or empirical models that describe ion cloud broadening specific to the IMS platform. Moreover, the algorithm may have applications in other scientific domains where deconvolution solutions for a set of prior experimental assumptions are required.

## Supplementary Material



## Notes

Further details on the algorithm and validation with synthetic ion mobility data; mass spectra; and deconvolution solutions for the different drift and collision voltages (PDF)
