# Supplementary material for: Deconvolution of High-Dimensional Ion Mobility Data Using Reversible-Jump Markov Chain Monte Carlo
Source: Anal Chem. 2026 Feb 26;98(9):6684–91. doi: 10.1021/acs.analchem.5c06427 (PMC12980490; doi:10.1021/acs.analchem.5c06427)
Supplement: Supplementary file 1 [file ac5c06427_si_001.pdf]

Supporting Information

Deconvolution of High-Dimensional Ion Mobility Data using Reversible-Jump Markov Chain Monte Carlo

Jerome Riedel[1,2], Marc Safferthal[1,2], Gergo Peter Szekeres[1,2], Kevin Pagel[1,2]

[1] Freie Universität Berlin, Altensteinstraße 23A, 14195 Berlin, Germany
[2] Fritz-Haber-Institut der Max-Planck-Gesellschaft, Faradayweg 4-6, 14195 Berlin, Germany

Table of Contents

1. *Sampling Constraints* ... *S1*
2. *Parameter Optimization* ... *S4*
3. *Equi-Energy Sampler* ... *S6*
4. *Reversible-Jump Markov Chain Monte Carlo (RJMCMC)* ... *S8*
5. *Bayesian Sequential Partitioning (BSP)* ... *S10*
6. *Instrument Parameters* ... *S12*
7. *Algorithm Parametrization* ... *S12*
8. *Algorithm Validation* ... *S13*
9. *Cytochrome c Mass Spectra* ... *S25*
10. *Deconvolution of Cytochrome c 7+ CCS Data* ... *S26*
11. *Deconvolution of Cytochrome c 7+ CIU Data* ... *S27*
12. *PGM O-Glycan measurements* ... *S28*
13. *References* ... *S31*

1. Sampling Constraints

In the context of Bayesian statistics in experimental chemistry/physics, prior probability distributions (priors) capture all the information that is available to the experimentalist at the time of conducting the experiment, and which may be required to derive a meaningful understanding of the measured data.[1] Finding deconvolution solutions of arrival time distributions (ATDs) requires knowledge about the transport properties of ions in the gas phase, i.e., the physical processes governing ion motion, such as diffusion and Coulomb repulsion. Ultimately, the ion-ion interactions define the statistical distribution (i.e., the expectation value and variance) that is observed when the ions arrive at the detector and hence determine derived properties such as intensity, signal-to-noise, etc.[2] For the deconvolution of ATDs, uninformative priors

were used as optimization boundaries during the deconvolution. These boundaries are expressed in the form of uniform probability distributions that assign equal probability of observation to each parameter, if the parameter stays in a pre-defined interval. Within the scope of this work, uninformative priors were used to model the minimum and maximum observable amplitude, the drift time interval where individual conformers can be observed, and the minimum and maximum separation distance between two conformers (see Figure S1). In some sense, the sampling of peak widths during the parameter optimization is an extension to the concept of priors, since the minimum and maximum peak widths are effectively limited to a predefined expectation of what the peak width should be for every arrival time and ion count. Based on variance contributions from ion injection $\sigma^2_{\mathrm{Inj.},t}$, diffusion $\sigma^2_{\mathrm{Diff.},t}$, and Coulomb repulsion $\sigma^2_{\mathrm{Coul.},t}$, the minimum peak variance for every arrival time *t* and number of ions N_i can be estimated by[2–4]

$$\sigma^2_t \geq \sigma^2_{\mathrm{min},t} = \sigma^2_{\mathrm{Diff},t} + \sigma^2_{\mathrm{Coul.},t} + \sigma^2_{\mathrm{Inj.},t}. \quad (1)$$

Depending on the complexity of the ATD and the operating principle of the ion mobility instrument, overlapping components and the sample background must be considered as additional sources of ion cloud broadening (e.g., no quadrupolar mass selection before the drift tube). Generally, the number of ions N_i in each component is given by

$$N_i = \frac{A_i}{A_{ATD}} N, \quad (2)$$

where N is the total number of ions under the ATD, A_i is the area of a single component and $A_{ATD}$is the total area under the ATD. However, in case of overlapping components or additional sample background, the value for N_i must be upscaled according to

$$N_i = \frac{A_{\mathrm{ATD.},n\sigma}}{A_{i,}} N_i,$$

where $A_{\mathrm{ATD.},n\sigma}$ is the area under the ATD in the $n\sigma$ confidence interval of the *i*-th component. Based on the arrival time t_i and ion count N_i of each component, the minimum, expected temporal ion cloud variance $\sigma^2_{\mathrm{min},t}$ provides an infimum below which ion cloud broadening cannot fall. To obey this limit, the exponential distribution was used to propose new peak widths after updates to the amplitude or arrival time of each component during the optimization. Based on the new amplitudes and number of ions, a multiple of the minimum ion cloud dispersion is sampled as $m = \frac{\sigma_t}{\sigma_{\mathrm{min.},t}}$ with probability

$$\Pr(m) = \begin{cases} 0, & m < 1 \\ \lambda \exp\left(-\lambda(m-1)\right), & \text{else.} \end{cases} \quad (3)$$

The parameter λ controls the steepness of the decline and is a hyperparameter suitable for optimization. Empirically, a value of $\lambda \approx 30$ has proven to yield reliable results in the deconvolution of reference ATD spectra and was also used in the deconvolution of the CCS and CIU spectra of cytochrome c. Generally, we found that large λ impacted the convergence performance of the algorithm, while small λ did not provide sufficient bounds on the peak width. From a standpoint of convergence during the Markov chain Monte Carlo procedure, it is sometimes useful to loosen this constraint slightly and choose shifts that are close to one. However, this was not used for the deconvolution of cytochrome c data and only for the *O*-glycan deconvolution as overall significantly less samples were drawn and convergence was reached faster.

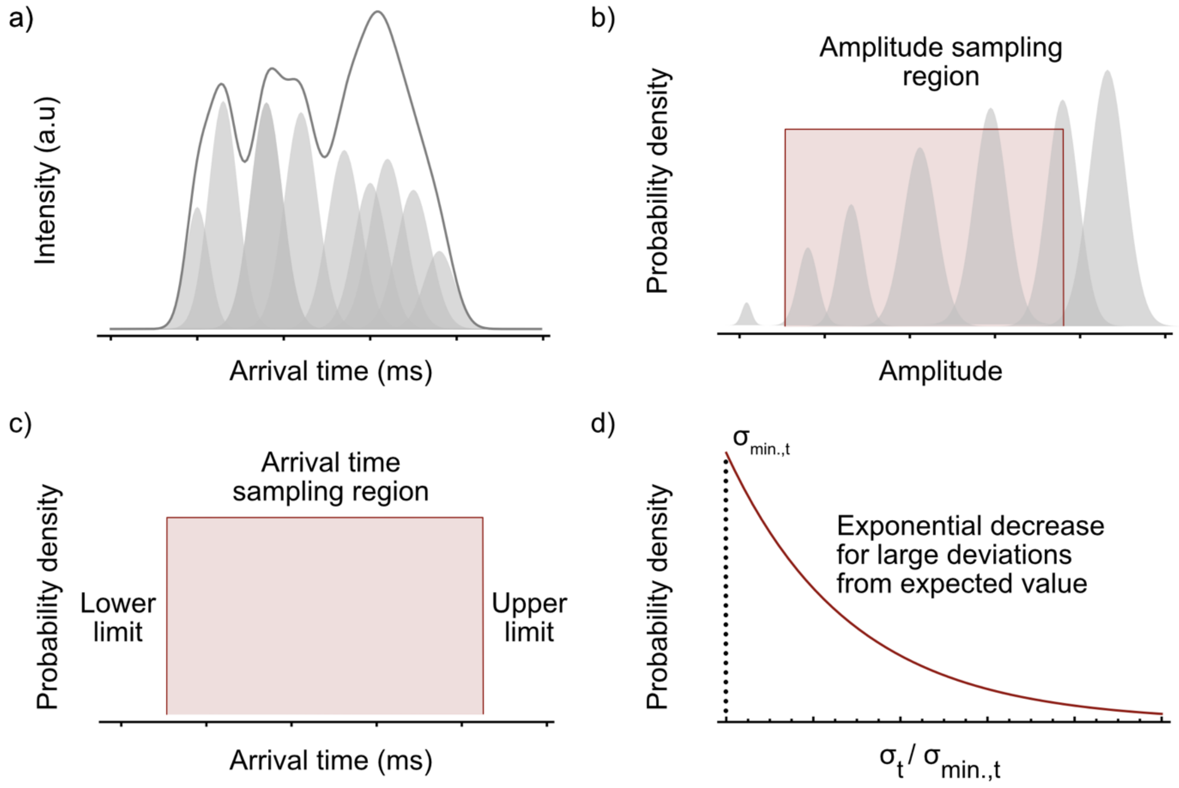

Figure S1. Illustrations of prior probability distributions for arrival time, amplitude, and related ion cloud broadening. a) Example of an arrival time distribution with multiple components. b) Uniform prior that is used to model the amplitude interval. The amplitude interval has direct physical limits derived from the number of detected ions. c) Uniform prior that was used to model the sampling interval for arrival times. The boundaries are inferred from the non-zero intensity region. d) Exponential distribution that was used to propose new ion variances based on the arrival time and number of ions. Large deviations from the expected minimum have a low probability of being sampled.

2. Parameter Optimization

Parameter optimization in the context of ATD deconvolution refers to the process of finding the parameters that best describe the experimentally observed data while staying consistent with expectations from ion transport theory. Then, the parameter vector of amplitudes A_i (ion count), peak centers μ_i (mean arrival time), and variance σ_i^2 (ion cloud spread) provides a reasonable explanation of the experimentally observed data *X* based on a Gaussian convolution model $M(t, \Theta)$

$$X = M(t, \Theta) = \sum_{i}^{k} \frac{A_i}{\sigma_i \sqrt{2\pi}} \exp\left(-\frac{(t-\mu_i)^2}{2\sigma_i^2}\right), \quad (4)$$

where t is the experimentally determined arrival time interval, Θ is the parameter vector, and the convolution is over the *k* components under the ATD.

Within the framework of Bayesian statistics, the goal is to calculate the posterior distribution $\Pr(\Theta \mid X)$ to infer the parameter vector Θ that best describes the experimentally observed data under a set of prior assumptions. According to Bayes' theorem, the posterior is given by[1,5]

$$\Pr(\Theta| X) = \frac{\mathcal{L}(\Theta| \mathrm{X}) \Pr(\Theta)}{\Pr(X)}, \quad (5)$$

where $\mathcal{L}(\Theta \mid \mathrm{X})$ is the likelihood of observing the parameter vector Θ given the observed data X, $\Pr(\Theta)$ is the prior, and $\Pr(X)$ is the evidence. Depending on the problem, the analytical calculation of the evidence $\Pr(X)$ is usually not possible, thus requiring the use of numerical methods to approximate the posterior through an invariant distribution (the invariant distribution is proportional to the posterior up to a normalization constant).[6] Directly sampling from the posterior, i.e., proposing new parameter vectors according to their observation probability can be achieved by Markov chain Monte Carlo (MCMC) techniques that rephrase the problem to the calculation of posterior ratios to avoid the calculation of the evidence.[6–8] Here, the posterior ratio refers to the probability ratio of observing two states with parameter vectors Θ and Θ', respectively. Evolving the chain for a sufficient number of iterations by altering the present state through a stochastic change, the collection of sampled states and their probability of occurrence provide an approximation to the posterior that eventually converges to the true posterior for an infinite number of samples up to a normalization constant. Independent of the MCMC algorithm used for sampling, the new state with parameter vector Θ' is directly derived from a stochastic variation

applied to the current state Θ and a generalized probability of acceptance to either move to that new state or to remain in the present state. The posterior ratio and the corresponding acceptance probability α for a move from $\Theta \rightarrow \Theta'$ are given in the Metropolis-Hastings formulation as[6]

$$\frac{\Pr(\Theta' \mid X)}{\Pr(\Theta \mid X)} = \frac{\mathcal{L}(\Theta' \mid \mathrm{X})\Pr(\Theta')}{\mathcal{L}(\Theta \mid \mathrm{X})\Pr(\Theta)}, \tag{6}$$

$$\alpha = \begin{cases} 1, & \text{if} \quad \dfrac{\Pr\left(\Theta' \middle| X\right)}{\Pr(\Theta \mid X)} \geq 1 \\ \mathcal{U}(0,1), & \text{else.} \end{cases} \tag{7}$$

To date, a variety of different implementations for MCMC have been formulated, which differ in the derivation of the new state Θ' to a large extent. Purely stochastic variations, as employed in the Random Walk Metropolis-Hastings (RWMH) algorithm, suffer from long iteration times as the evolution of the chain is undirected and multi-modal posteriors pose a difficult challenge. To mitigate these problems, simulated annealing algorithms have been developed to escape local minima by varying the “temperature” of the system across the iteration time, where at high temperatures efficient exploration of the full sample space is guaranteed and at low temperatures solutions are refined.[7,9–11] Overall, these algorithms have higher chances of finding the global solution.[7]

Finding solutions for the deconvolution of ATD data using MCMC techniques requires the formulation of a likelihood function $\mathcal{L}(\Theta \mid \mathrm{X})$. For data fitting algorithms, the likelihood function is directly related to the fitting error, which has an inherent lower limit due to deviations from a perfect Gaussian peak shape description in case of DT-IMS. In practice, each time point t_i with observed intensity y_i in the ATD has an associated normal distributed noise ϵ_i. Given the standard deviation of the noise σ_n, a likelihood function is then given by[12,13]

$$\mathcal{L}(\Theta \mid \mathrm{X}) = \prod_{i=1}^{N} \mathcal{N}(y_i - M(t_i, \Theta) \mid 0, \sigma_n), \tag{8}$$

where $\mathcal{N}$ is the normal distribution centered at 0 with standard deviation σ_n. Along with the priors specified (see Sampling constraints), MCMC schemes will try to infer the posterior through continuous application of a stochastic change. The approach to derive the stochastic change, either static or dynamic, differentiates MCMC schemes and eventually determines their convergence properties.

3. Equi-Energy Sampler

For a successful deconvolution of high-dimensional ATD data, it is important to use algorithms that generate solutions that are agnostic to the initial starting conditions but still have high probability of converging into the global solution. In the past, different optimization strategies for global solutions have been proposed. Here, the category of simulated annealing algorithms has shown great potential as they are routinely applied in statistical optimization problems and machine learning.[5,7,12] In the case of the equi-energy sampler, good convergence properties have been demonstrated for multimodal distributions and complicated inference problems.[9] With a statistical-mechanics-motivated energy function that is proportional to the posterior

$$\mathrm{Pr}(\Theta\,|X) \propto \exp\left(-h_T(\Theta)\right), \tag{9}$$

the energy function $h(\Theta)$ serves as a gateway to overcome local trapping phenomena by connecting it to an artificial temperature T. N Markov chains at different temperatures are then evolved in parallel, while samples can be transferred between successive Markov processes based on their energy.

In the energy formalism, the complete sample space of each chain is mapped to an energy surface. Furthermore, the complete energy surface of every chain can be divided into energy truncations

$$H_1 < H_2 < \cdots < H_N < H_{N+1} = \infty, \tag{10}$$

where each chain has an associated temperature T_i such that

$$1 = T_1 < T_1 < T_2 < \cdots < T_N. \tag{11}$$

For each energy truncation $[H_i, H_{i+1})$ at temperature T_i, a distribution $\mathrm{Pr}_i(\Theta\,|X)$ targeted by a Markov chain with energy function $h_i(\Theta) = \frac{1}{T_i}\max(h(\Theta), H_i)$ is defined. Each Markov chain then samples from h_i, thus guaranteeing efficient sampling of the complete parameter space at high temperatures (distribution flattening), while at $T_1 = 1$, the samples will be directly drawn from the posterior $\mathrm{Pr}_{\,i=1}(\Theta_\mathrm{k}\,|X)$. The higher-temperature chains are explicitly introduced for mixing with lower-temperature chains to overcome local energy barriers. With equi-energy jump probability p_{ee}, an equi-energy sample from the invariant distribution $\mathrm{Pr}_{i+1}(\Theta\,|X)$ can be drawn to allow a lower-temperature chain to explore higher-energy sampling regions. The acceptance probability α of the equi-energy jump is given by[7,9]

$$\alpha = \min\left(1, \frac{\exp\left(h_k\left(\Theta_{j+1}^{(i+1)}\right)\right)\exp\left(h_{k+1}\left(\Theta_j^{(i)}\right)\right)}{\exp\left(h_k\left(\Theta_j^{(i)}\right)\right)\exp\left(h_{k+1}\left(\Theta_{j+1}^{(i+1)}\right)\right)}\right), \quad (12)$$

where $\Theta_j^{(i)}$ is the current sample in the lower-temperature chain i and $\Theta_{j+1}^{(i+1)}$ is the proposed next state at $j+1$ drawn from the equi-energy ring of the higher temperature chain $i+1$. If the jump is not accepted, the present state is not updated. If the probability for an equi-energy jump is not reached, the momentary state undergoes a classical Metropolis-Hastings (MH) move. In summary, the sampling process starts with the highest-temperature chain that is evolved using classical MH moves. After a burn-in period, each sample is assigned to the respective energy ring based on the current energy of the sample. The complete sample space $\mathcal{S}$ can then be represented by the union of all energy sets $\mathcal{S} = \bigcup_{i=1}^{M} D_i$ where $D_i = \{\Theta : h(\Theta) \in [H_i, H_{i+1})\}$. Following the sampling process of the highest-temperature chain, the next highest-temperature Markov process is started, which can now undergo local- and equi-energy updates by drawing equi-energy samples directly from the higher-temperature chain. The process is repeated, until the lowest temperature process has finished, which directly samples from the target posterior.

To guarantee efficient sampling of the complete parameter space, recommendations for the setup of energy rings, temperatures, and update distributions in the local MH move have been proposed.[9] At the beginning, energy values for H_0 and H_K must be found. For most problems, it is convenient to directly link the energy function to the likelihood and prior by $h(\Theta) = -\mathcal{L}(\Theta \mid \mathrm{X}) \Pr(\Theta)$. Hence, H_0 can be determined for the perfect fit of the observed data, i.e., without error in the fitted data points and perfect agreement with the model constraints. Approximations to H_K can be found for poor descriptions of the observed data, which is generally the case for the non-biased selection of initial starting parameters. Thus, setting H_K to the energy of the initial parameters provides a good guess to the non-trivial upper energy limit. For the energy limits in-between, it has been shown that a geometric progression provides well-distributed energy rings. Temperatures can be inferred from the energy distance in each interval and can be approximated by $T_i \approx (H_{i+1} - H_i)/c$ where $c \in [1, 5]$. For ATD deconvolution, we have found that setting $c \in [0.1, 3]$ yields temperatures that later integrated well with the temperature-dependent stochastic variations for each parameter. Based on the obtained temperatures, we can define appropriate MH

transition kernels for each chain based on the standard deviation $\sigma_s^{(1)}$ of the lowest-temperature process. For each chain i at temperature T_i, the stochastic change can be drawn from a normal distribution with standard deviation $\sigma_s^{(i)} = \sigma_s^{(1)} \sqrt{T_i}$. For the equi-energy jump probability, values for p_{ee} between 1.5 – 5% have shown good results. Throughout this work, a value of p_{ee} = 1.5% was used.

4. Reversible-Jump Markov Chain Monte Carlo (RJMCMC)

The first generalization of MCMC techniques to multiple dimensions was given by Green in 1995.[14] In RJMCMC, the invariant distribution over a finite or infinite number of models is calculated with the goal to select the model with highest posterior probability (model selection). Detailed discussions of the technical intricacies have been given elsewhere[14–16] and, in the following, only the most important aspects are given regarding the setup of the RJMCMC algorithm and feasible implementations for ATD deconvolution.[14–17]

Given a countable set of models $\{M_1, \ldots, M_k\}$, RJMCMC is centered around the problem of joint inference of the model indicator k and the corresponding parameter vector Θ_k, i.e., calculating the joint posterior $\Pr(k, \Theta_k|X)$ for the experimentally observed data X according to[14]

$$\Pr(k, \Theta_k|X) \propto \Pr(k|\mathrm{X}) \Pr(\Theta_k|k, X), \tag{13}$$

where $\Pr(\mathrm{k}, \Theta_k|\mathrm{X})$ and $\Pr(\Theta_\mathrm{k}|k, X)$ are the model- and parameter specific posteriors, respectively. Note that the transition from $\Theta \to \Theta_k$ is made in this case to explicitly highlight the dimension of the parameter vector but is otherwise mathematically equivalent to Θ in the previous section. In case of the ATD deconvolution problem, each model M_k represents a fixed number of components that potentially describes the observed ATD data through the parameter vector $\Theta_k \in \mathbb{R}^{3k}$. Since the analytical calculation of the posterior distributions are generally not tractable, the joint posterior can be approximated by invariant distributions obtained through numerical calculations. Since sampling from different parameter spaces can involve complex transformations and varying dimensions, a generalized Markov chain formalism is required. This formalism is usually introduced through the concept of a trans-dimensional jump function that ensures detailed balance, i.e., consistent acceptance probabilities for the forward and backwards move across dimensions.[15,16] For this generalized Markov process, a jump function j maps the current state (Θ_k, u) to a new

state $(\Theta_{k'}, u')$ using the auxiliary variables u and u'.[16] Depending on the complexity of the jump function, calculating the required derivatives for jumps between different dimensions in the classical RJMCMC formulation can be a cumbersome task, which is further complicated by the fact that theory-motivated jump functions are not always accessible. To circumvent the formulation of a jump function, “gold chain” simulations have evolved in recent years as an easy-to-use approach to RJMCMC.[17] Here, individual fixed-dimensional posteriors are used to sample jumps to higher- or lower-dimensional parameters directly from the dimension higher or -lower posterior, respectively. Using Bayes’ formula, the dimension posterior distribution $\mathrm{Pr}(k, \Theta_k|\mathrm{Y})$ can be expressed as follows[17]

$$\mathrm{Pr}(k, \Theta_k|X) = \frac{\mathrm{Pr}(X|k)\,\mathrm{Pr}(k)}{\mathrm{Pr}(X)}, \tag{14}$$

with the fixed-dimension parameter posterior given by[17]

$$\mathrm{Pr}(\mathit{\Theta}_k|\,X) = \frac{\mathrm{Pr}(X|\mathit{\Theta}_k)\,\mathrm{Pr}(\mathit{\Theta}_k)}{\mathrm{Pr}(X|k)}. \tag{15}$$

Rearrangement of equation (*15*) and substitution into equation (*14*) yields the following expression for the dimension posterior[17]

$$\mathrm{Pr}(k, \Theta_k|X) = \frac{\mathrm{Pr}(X|\Theta_k)\,\mathrm{Pr}(\mathit{\Theta}_k)\,\mathrm{Pr}(k)}{\mathrm{Pr}(X)\,\mathrm{Pr}(\mathit{\Theta}_k|X)}, \tag{16}$$

with the evidence $\mathrm{Pr}(X)$ being the only unknown quantity. According to equation (*16*), the acceptance probability for jumps from a parameter vector Θ_k to $\Theta_{k'}$ is then given by

$$\alpha(\Theta_k, \Theta_{k'}) = \min\left(1, \frac{\mathrm{Pr}(X|\Theta_k)\,\mathrm{Pr}(\Theta_k'|X)\,\mathrm{Pr}(\mathit{\Theta}_k)\,\mathrm{Pr}(k)}{\mathrm{Pr}(X|\Theta_{k'})\,\mathrm{Pr}(\mathit{\Theta}_k|X)\,\mathrm{Pr}(\mathit{\Theta}_k')\,\mathrm{Pr}(k')}\right). \tag{17}$$

For the derivation of equation (17) as stated here, equal probability for the forward and backward move was assumed. Based on equation (*17*), it is now evident that, instead of proposing new parameter vectors from a jump function, samples can be drawn from the fixed-dimensional posteriors that are accessible through classical MCMC simulations, which are then weighted by the likelihood, parameter-, and dimension prior for the respective dimension. Therefore, the problem of proposing new jumps is shifted towards the accurate determination of the posterior probability from the posterior samples obtained in the classical MCMC step, which are then used as one-to-one mappings to perform jobs between regions of similar density.

5. Bayesian Sequential Partitioning (BSP)

In the gold-chain formulation of RJMCMC, it is crucial to find a numerical approximation to the invariant distribution obtained from the fixed-dimensional sampling runs. Due to the high dimensionality of the parameter space, it is important to use algorithms that maintain quality of estimation in moderately large (< 50 variables, i.e., 10-20 Gaussian peaks) sample spaces. Bayesian sequential partitioning was derived as a local density adaptive algorithm that is agnostic to the underlying distribution of data. In the following, a description of the algorithm and the computational implementation of earlier works is given.[18,19]

Given the sample space $\mathcal{S}$ after the fixed-dimensional MCMC run with samples $\Theta_i = \left(A_{i,1}, \mu_{i,1,}\sigma_{i,1}^2, \dots, A_{i,k}, \mu_{i,k,}\sigma_{i,k}^2\right) = (z_{i,1}, \dots, z_{i,3k})$ where $\Theta_\mathrm{i} \in \mathbb{R}^{3k}$, the measurable space $(\mathcal{S}, \Sigma)$ is defined. For simplicity, $\mathcal{S}$ is represented as a bounded rectangle defined by $\mathcal{S} = I_1 \otimes I_2 \otimes \dots \otimes I_{3N}$ with $I_j = \left(\min(z_{i,j}), \max(z_{i,j})\right)$. The σ-algebra Σ is defined as the collection of subsets obtained via recursive binary partitioning, i.e., a cut along the midpoint of a dimension followed by accumulating all samples that are located in the newly created hyperrectangle, such that $\Sigma = \{A_1, \dots, A_t\}$, where A_i is a subregion from cutting and $t - 1$ is the number of cuts. A *simple* function f is defined on the σ-algebra as [18]

$$f(\Theta) = \sum\nolimits_{i=1}^{t} \frac{\theta_i}{|A_i|} I_{A_i}(\Theta), \qquad (18)$$

where $I_{A_i}(\Theta)$ is the indicator function that evaluates to 1 if $\Theta \in A_i$, else 0, $\theta_i = \Pr(\Theta \in A_i)$ is a Dirichlet-distributed variable with parameters $(\alpha, \dots \alpha)$ and $|A_i|$ is the volume of the i-th subregion. Here, θ_i captures the prior information about the distribution of individual parameter vectors to each subregion. The Dirichlet parameter α is chosen as a constant with no *a priori* bias about the distribution of samples in one of the subregions. Since multiple cuts and cut histories are possible for the approximation of the density, an objective descriptor for the quality of the partition was defined via Bayesian statistics as[18]

$$\Pr(A_1, \dots, A_t | D) \propto \Pr(D | A_1, \dots, A_t) \Pr(A_1, \dots, A_t). \qquad (19)$$

By integration over every sample in every subregion according to the Dirichlet distribution $\mathrm{Dir}(\alpha)$,[20] the posterior density can be accumulated based on equation (18) as[18]

$$\Pr(A_1, \dots, A_t|D) \propto \Pr(A_1, \dots, A_t) \int \left(\prod_{i=1}^{t} \left(\frac{\theta_i}{|A_i|} \right)^{n_i} \right) \mathrm{Dir}(\alpha) d\theta_1 \dots d\theta_t, \tag{20}$$

where n_i is the number of samples in the i-th subregion. Furthermore, the prior over the number of subregions $\Pr(A_1, \dots, A_t)$ is represented by an exponential distribution $\exp(-\beta t)$ that reflects the exponentially decreasing probability of observing the t-th subregion. Based on equation (20), the partition score is obtained after integration as the logarithm of the posterior such that[18,19]

$$s(P_m) = -\beta t + \log\left(\frac{B(n_1 + \alpha, \dots, n_t + \alpha)}{B(\alpha, \dots, \alpha)} \right) - \sum_{i=1}^{t} \mathrm{n_i} \log(|A_i|), \tag{21}$$

where P_m is the m-th partition. Exploring the full set of partitions is computationally exhaustive and grows exponentially in time complexity. Thus, approximations were developed that allow for an efficient sampling of a representative number of subregions.[19] Given an initial set of *M* partitions, every partition explores all possible cuts in all subregions that are available to that point. Based on a partition score, the cuts are evaluated with respect to their ability to distribute samples to one of the halves created by the cut line. Here, cuts with high asymmetry, i.e., where most of the points are located in one of the newly created subregions, are incentivized. The probability of observing a cut is given by[19,21]

$$s_{j_{id}}\left(c_{j_{id}} \middle| \{A_1, \dots, A_{j-1})\right\} = C_{j-1}\, 2^{n_i} \frac{\Gamma\left(n_{id}^{(1)}\right)\Gamma\left(n_{id}^{(2)}\right)}{\Gamma(n_i)} \tag{22}$$

where $s_{j_{id}}$ is the conditional probability of executing the cut $c_{j_{id}}$ at level *j* along dimension *d* in the i-th subregion with n_i samples, $n_{id}^{(1)}$ and $n_{id}^{(2)}$ are the respective numbers of samples in the left and right halves of the newly created subregions after the cut, Γ is the Gamma function,[22] and C_{j-1} is a normalization constant. This process is repeated until a maximum number of cuts has been reached or the partition score converged. Finally, the best partition score is chosen according to equation (*21*).

The discretized posterior can be calculated from the best posterior by evaluating the density in every subregion according to $\frac{n_i}{N|A_i|}$ where N is the total number of samples subjected to the BSP algorithm.

In agreement with the empirical findings*,* the BSP algorithm as outlined here was initialized with at least 100 partitions and a convergence threshold of 30 consecutive cuts that did not result in an improvement of the partition score. A maximum number

of 300 cuts was explored. The computational implementation of equations (*21*) and (22) uses the C++ library Symengine for symbolic computation of the Gamma- and power function.[23]

6. Instrument Parameters

In this work, a modified Synapt G2-S instrument was used that was described in detail previously.[24] The original IMS cell was replaced by a 25.05 cm drift tube consisting of a set of stacked ring electrodes in a radio frequency confining electric field. Ions are injected into the drift tube with a rectangular injection pulse of 100-200 µs. The instrument can be operated with nitrogen and helium buffer gas, and the pressure is regulated by a 647C multi gas controller (MKS Inc., Andover MA, United States) that is connected to a 627H capacitance manometer (MKS Inc., Andover MA, United States). The temperature of the instrument is monitored with a standard PT100 resistance thermometer that is mounted on the drift tube housing. During the measurements, the temperature was between 297-298 K.

Measurements of cytochrome c were conducted at 1.8 torr in helium, while all *O*-glycans were measured in nitrogen at 2.2 torr to achieve higher electric field strengths and increase comparability with measurements on a Bruker timsTOF Pro.

7. Algorithm Parametrization

For equi-energy sampling, the lowest achievable energy H_1 was set to the energy-equivalent of a perfect ATD description given by the negative likelihood at zero deviation between deconvolution and experiment. For the upper energy limit H_K, the energy of the starting parameters was used. The starting parameters are generally a very poor guess of the solution and are therefore high in energy. Subsequent energy limits were then determined from a geometric progression in the interval $[H_1,\ H_K]$. The number of chains K was set to 150. The temperature of each chain was determined according to $T_i = (H_{i+1} - H_i)/c$ where $c \in [1,3]$ was used in the deconvolution of synthetic and cytochrome c ATDs. In case of the *O*-glycan samples, it was beneficial to increase the temperature by setting $c \in [0.1, 1]$. The step size σ_i of each chains' MH transition kernel was scaled with $\sigma_s\sqrt{T_i}$, where σ_s was inferred from the ATD data. If preliminary tests showed a high-dimensional ATD, i.e., more than 4 components under the ATD, each chain was started with a burn-in period of $5 \text{ x } 10^4$ samples and the total

number of samples in each chain was set to 1×10^6. For lower-dimensional problems the number was reduced to 5×10^3 burn-in samples and 5×10^4 samples. The equi-energy jump probability was set to 1.5 %. Experimental data of cytochrome c and *O*-glycans were measured without quadrupole mass selection, and the ion background was considered in the 1σ and 0.25 σ confidence interval of each component, respectively. We found that a smaller confidence interval gives a better agreement with the experiment if the ion background is substantially higher than the ion count of the species of interest. The charge density was modeled assuming a uniform distribution ($C_{\mathrm{Coul.}}$ = 1/3). After equi-energy sampling, a classical MH sampler with a narrower proposal distribution around smaller steps were used to refine the posterior samples. For the posterior density estimation using BSP, 100 partitions were evolved for a maximum of 400 cuts. The convergence threshold was set to 30 for consecutive improvements of the partition score. The posterior mean was evaluated on the last 3×10^4 samples of the equi-energy stage. Given the discretized posteriors, RJMCMC runs with a burn-in phase of 5×10^4 samples and 5×10^6 total samples were performed. The posterior over the model dimension is reported as the percentage of samples in the given dimension divided by the total number of samples.

8. Algorithm Validation

Before using the algorithm on experimental data, the performance was validated on synthetic data. In the following, the abbreviation *m*c → *n*c will be used to describe the notion of fitting *m* components to an *n*-component ATD. The algorithm was validated on artificial data that was modeled with randomly assigned fractions of the total ion count for ATDs with fixed arrival times of 5, 7, and 9-components. The injection time, drift voltage, drift tube length, and temperature were set to 200 μs, 62.3 V, 0.2505 m, and 298 K, respectively, which is comparable to the experimental drift tube conditions used in this work. The total number of ions subjected to the ATD was 10^5, each with a charge of 7+. During the fixed-dimensional sampling stage, equi-energy sampling was performed for models that deviate from the reference value by -1 to +2 components. The initial peak centers were obtained from evenly distributing the components across the nonzero intensity region of the ATD.

A special emphasis in the discussion of the algorithm performance is put on the 9-component deconvolution as generally believed to be the most challenging of the test set.

Across all *m*c → *n*c deconvolution solutions low root-mean-square errors (RMSE, < 0.5) from an uninformed guess of parameter locations are obtained (see Table S1) after equi-energy sampling.

Table S1. RMSE of deconvolution models after equi-energy sampling.

n	*n*c → 5c	*n*c → 7c	*n*c → 9c
4	5.02	-	-
5	0.13	-	-
6	0.16	0.31	-
7	0.17	0.15	-
8	-	0.17	0.19
9	-	0.15	0.19
10	-	-	0.19
11	-	-	0.19

The only case where convergence could not be reached was the 4c → 5c deconvolution. As anticipated, based on the observed RMSEs across the different *m*c → *n*c deconvolutions, with increasing number of components, the quality of deconvolution can be maintained. As the number of components increases, ion counts are transferred to additional components to ensure that the total ion count is preserved. This preservation is crucial, as Coulomb repulsion plays a significant role in high-charge-state molecular ions. Generally, the lowest RMSE is observed for the true number of components, while other-dimensional deconvolutions exhibit slightly higher RMSE. This suggests that the rigidity of model constraints effectively mitigates the risk of under- and overfitting. Consequently, discriminating against higher- or lower-dimensional models in the RJMCMC step based solely on RMSE should be possible. A summary of the results after equi-energy sampling for the 5c → 5c, 7c → 7c, and 9c → 9c deconvolution is given in Table S2 - Table S4.

Table S2. Deconvolution results for 5c → 5c. Parameters are reported in order as reference parameters, optimized peak parameters, and (starting parameters). Optimized peak amplitudes accumulate to a total ion count of 10^5 after multiplication with the reference ion count (10^5).

	Amplitude A_i (a.u)	Arrival time μ_i (ms)	σ_i (ms)	Ion count
**1**	0.25 0.25 (0.05)	7.60 7.60 (7.18)	0.18 0.18 (0.26)	25000
**2**	0.25 0.25 (0.05)	8.30 8.30 (7.79)	0.20 0.20 (0.28)	25000
**3**	0.10 0.09 (0.05)	8.70 8.70 (8.40)	0.16 0.16 (0.30)	10000
**4**	0.15 0.15 (0.05)	9.20 9.20 (9.01)	0.19 0.19 (0.32)	15000
**5**	0.25 0.25 (0.05)	9.70 9.70 (9.62)	0.23 0.23 (0.34)	25000

Table S3. Deconvolution results for 7c → 7c. Parameters are reported in order as reference parameters, optimized peak parameters, and (starting parameters). Optimized peak amplitudes accumulate to a total ion count of 10^5 after multiplication with the reference ion count (10^5).

	Amplitude A_i (a.u.)	Arrival time μ_i (ms)	σ_i (ms)	Ion count
**1**	0.02 0.02 (0.05)	8.00 8.01 (7.83)	0.11 0.12 (0.28)	2000
**2**	0.18 0.18 (0.05)	8.30 8.30 (8.26)	0.18 0.19 (0.30)	18000
**3**	0.20 0.20 (0.05)	8.60 8.60 (8.69)	0.19 0.18 (0.31)	20000
**4**	0.10 0.010 (0.05)	9.20 9.20 (9.12)	0.17 0.18 (0.33)	10000
**5**	0.10 0.05 (0.05)	9.70 9.66 (9.55)	0.18 0.19 (0.34)	10000
**6**	0.15 0.20 (0.05)	10.00 9.94 (9.99)	0.21 0.20 (0.36)	15000
**7**	0.25 0.25 (0.05)	10.30 10.29 (10.41)	0.24 0.24 (0.37)	25000

Table S4. Deconvolution results for 9c → 9c. Parameters are reported in order of reference parameters and optimized parameters after equi-energy sampling. Relative amplitudes add up to a total ion count of 9.98 x 10^4 after multiplication with the initial ion population of 10^5 ions.

	Amplitude A_i (a.u.)	Arrival time μ_i (ms)	σ_i (ms)	Ion count
**1**	0.060 0.053	8.00 7.99 (7.73)	0.14 0.14	6000
**2**	0.140 0.151	8.30 8.30 (8.10)	0.17 0.17	14000
**3**	0.150 0.133	8.80 8.79 (8.48)	0.18 0.18	15000
**4**	0.150 0.163	9.20 9.19 (8.85)	0.19 0.19	15000
**5**	0.125 0.144	9.70 9.73 (9.23)	0.19 0.20	12500

**6**	0.100 0.090	10.00 10.03 (9.60)	0.19 0.17	10000
**7**	0.125 0.123	10.20 10.23 (9.98)	0.20 0.22	12500
**8**	0.100 0.075	10.50 10.49 (10.35)	0.20 0.17	10000
**9**	0.050 0.066	10.80 10.77 (10.73)	0.18 0.19	5000

Deconvolution solutions for *m*c → 9c are shown Figure S2. Good agreement is observed between the reference- and optimized parameters for peak centers with a mean error of 0.1% across all components. Medium deviations are observed for peak amplitudes and -widths with mean (absolute) errors of 0.4% (4%) and 0.6 % (6%), respectively. Here, the deviation originates from minor ambiguities in the assignment of ion counts for the different components. In summary, for all tested deconvolutions *m*c → *m*c, the equi-energy sampler is successful to either fully extract the original reference parameters (5c → 5c and 7c → 7c, see Table S2 and Table S3) or come qualitatively close in the case of the 9c → 9c deconvolution (see Table S4). In case of the 9c → 9c deconvolution, the original reference parameters in the first half of the ATD (< 9.7 ms) are successfully inferred, but above 9.7 ms, the ATD signal shape provides no additional information on peak widths due to an unresolved, broad, uniform peak. Here, the number of conformer ions can be arbitrarily assigned between neighboring conformers without loss of deconvolution quality; however, the qualitative amplitude structure, i.e. relative population levels, are maintained. Therefore, ATD deconvolutions with more than 7-conformers must not be overanalyzed when it comes to the extraction of absolute population levels as in regions of broad uniform intensity their assignment might be ambiguous (see also Figure S4 and Figure S5).

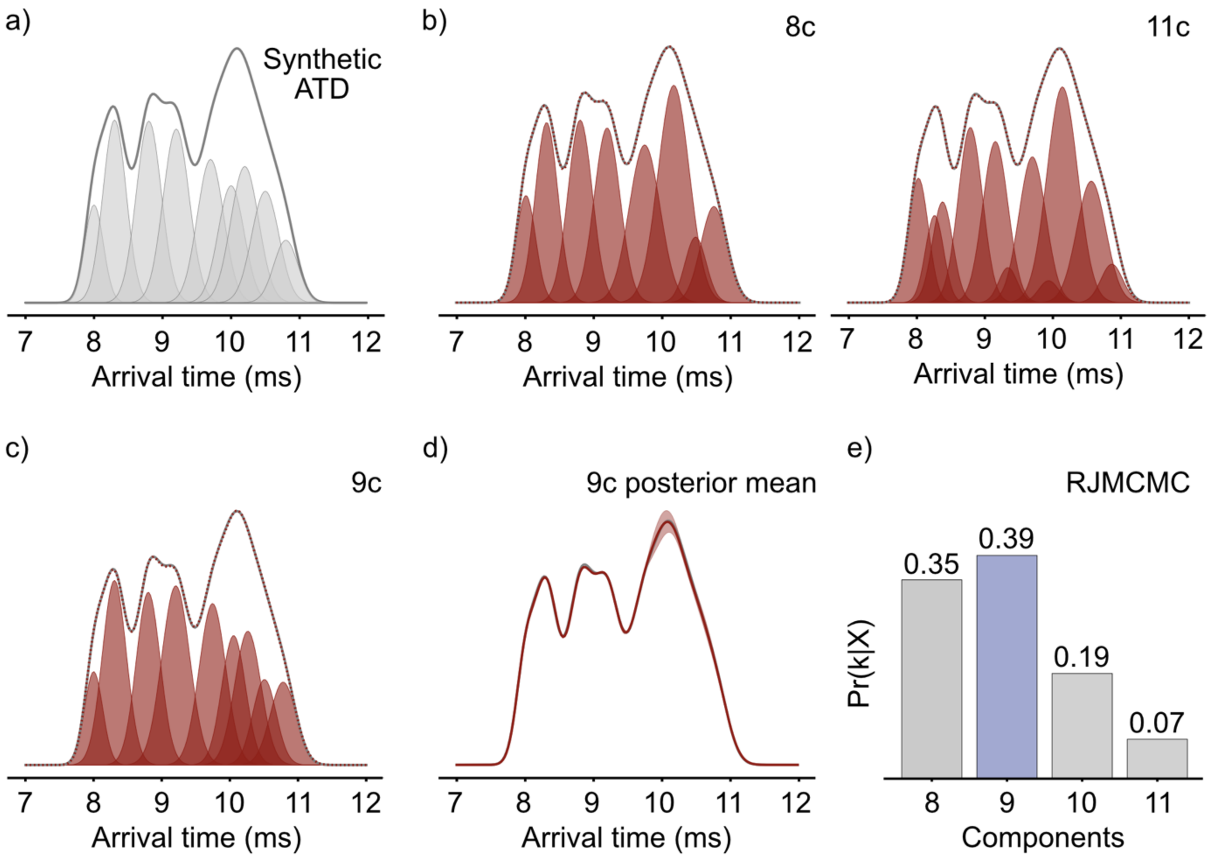

Figure S2. Applied RJMCMC workflow for multi-component ATDs. a) Synthetic 9-component ATD for simulated arrival times and ion count. b) Equi-Energy deconvolution solutions for fitting 8c (underfitting) and 11c (overfitting) to the 9-component synthetic ATD. Quality of deconvolution can be maintained by grouping or splitting individual components. c) 9c → 9c deconvolution solution after equi-energy sampling. d) Discretized BSP posterior based on the samples after 9c equi-energy sampling. The posterior mean after drawing 5 x 10^4 random variates is shown in red along with the 3σ confidence interval. e) RJMCMC calculates the probability for all tested components counts (8c – 11c). The overall highest probability was observed for the reference count of 9 components.

After equi-energy sampling, the different sampled model parameters were subjected to the BSP algorithm to calculate a discretized posterior for later use in the RJMCMC algorithm. Convergence of the algorithm is reached in all cases in < 40 binary partitions. Based on the BSP results, a representation of the posterior can be readily obtained by drawing random variates from each subregion according to the subregion density (see Figure S2). From the reconstructed ATD it can be immediately seen that different regions in the ATD, and thus different components, show different levels of confidence in their determined peak amplitudes, -centers, and -widths. Below arrival times of 10 ms, the confidence in the deconvolution solution is high. This is in good

agreement with the analysis of the equi-energy results of the synthetic data, where the original reference parameters are fully inferred during the optimization in informative areas of the ATD (8 – 10 ms). Above 10 ms, the confidence interval covers a higher-intensity band, which is a direct reflection of different levels of peak amplitudes and -widths that can be assigned to each component. Similar results are observed in the BSP stages for 5- and 7-components, where the confidence is generally lowest around areas of broad, uniform intensities (see Figure S6 and Figure **S7**). Hence, expectations about the correctness of population levels can be adjusted based on the spread of the confidence interval.

After conversion of the equi-energy results into a BSP-discretized posterior, each posterior can be used in the RJMCMC scheme for automatic determination of the number of components under the ATD. Since the RMSE difference between the tested model dimensions is small, it is expected that the uncertainty in predicting the correct number of components is high. Therefore, long iteration times in the RJMCMC stage are required to confidently approximate the posterior mean over the model dimension. The result after RJMCMC for the $m\text{c} \rightarrow 9\text{c}$ deconvolution set is shown in Figure S2e. The posterior mean over the model dimension is 9.0, which is equivalent to the number of components that was initially used to simulate the ATD. However, the variance of the posterior is large, which leads to a very uniform distribution of probabilities across the number of components with the majority of the probability being assigned to 8- and 9-components. Discriminating against the 8-component deconvolution is difficult, since the difference between the $8\text{c} \rightarrow 9\text{c}$ and $9\text{c} \rightarrow 9\text{c}$ deconvolution is routed in the single merger of two neighboring components around 10.2 ms into one larger species to which all neighboring components accommodate. Higher confidence values after RJMCMC are generally reached for the set of $m\text{c} \rightarrow 5\text{c}$ and $m\text{c} \rightarrow 7\text{c}$ deconvolutions (see Figure S3) that already show a larger difference in RMSE after the equi-energy stage. Here, the posterior mean is predicted to 5.5 and 7.0, respectively, with an overall more pronounced probability to choose the correct model dimension (55 % for 5c and 58 % for 7c). More importantly, the

RJMCMC characteristically assigns higher confidence to lower dimension models, which is preferred from a standpoint of overfitting.

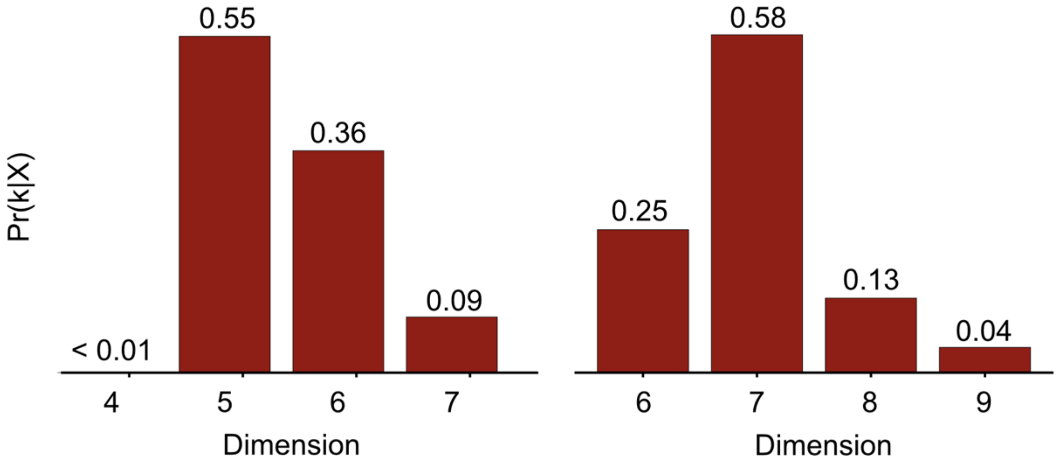

Figure S3. The dimension posterior for the inference of the 5- (left) and 7-conformer (right) mixtures after RJMCMC. The average dimensions are 5.5 and 7.0, respectively. As shown in Figure S6 and Figure S7, all posteriors yield good estimates of the ATD, thus uncertainty for the true model dimension is high.

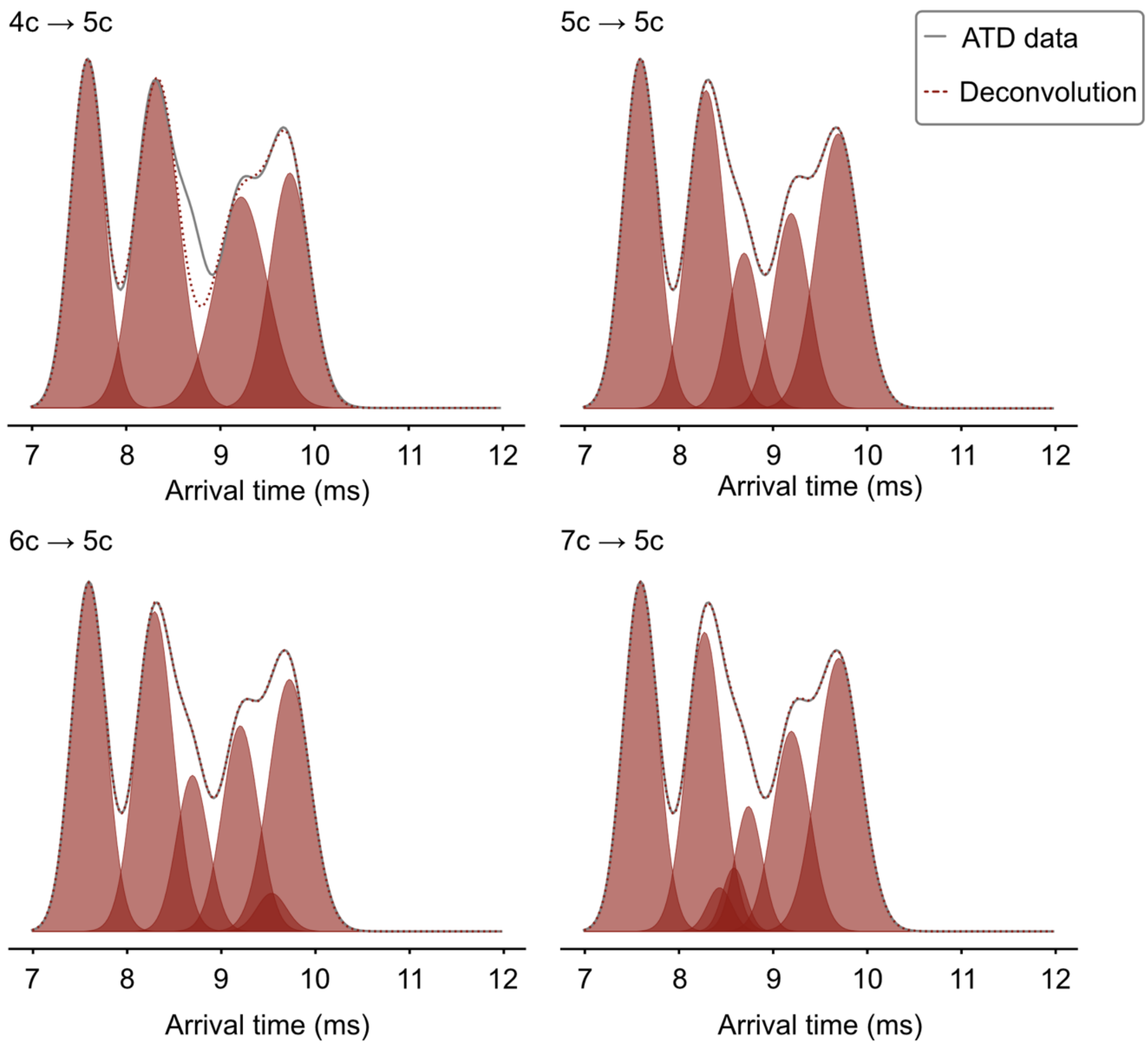

Figure S4. Deconvolution solutions after equi-energy sampling for all probed *m*c → 5c dimensions. The 4c → 5c deconvolution was not successful. Increases in dimension can always be reached by splitting high-intensity components into two lower-intensity components.

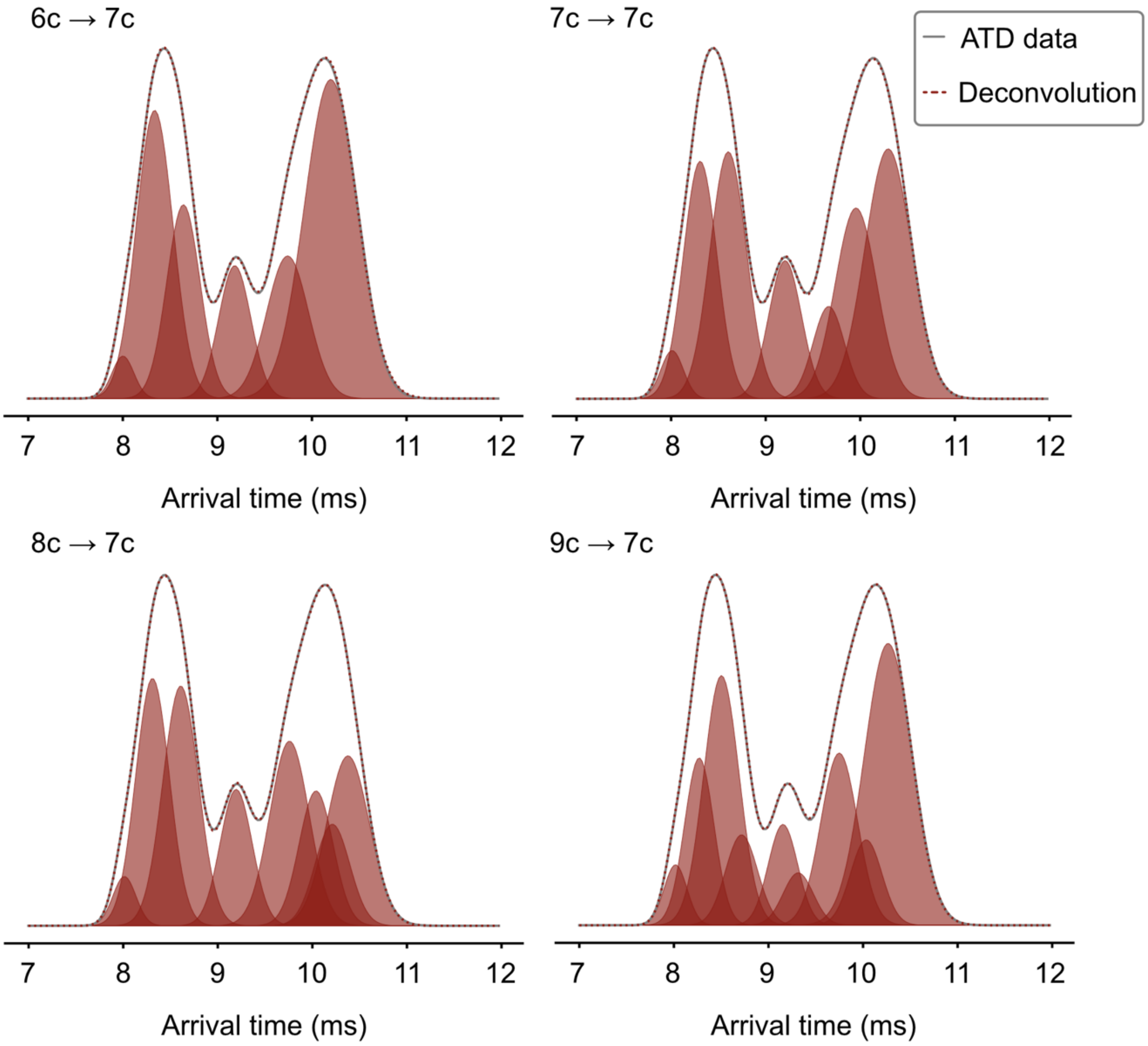

Figure S5. Deconvolution solutions after equi-energy sampling for all probed *mc* → 7c dimensions. All deconvolutions yield low RMSE solutions. For the 7c → 7c, a full qualitative recovery of initial parameters and relative peak amplitude ratios was found.

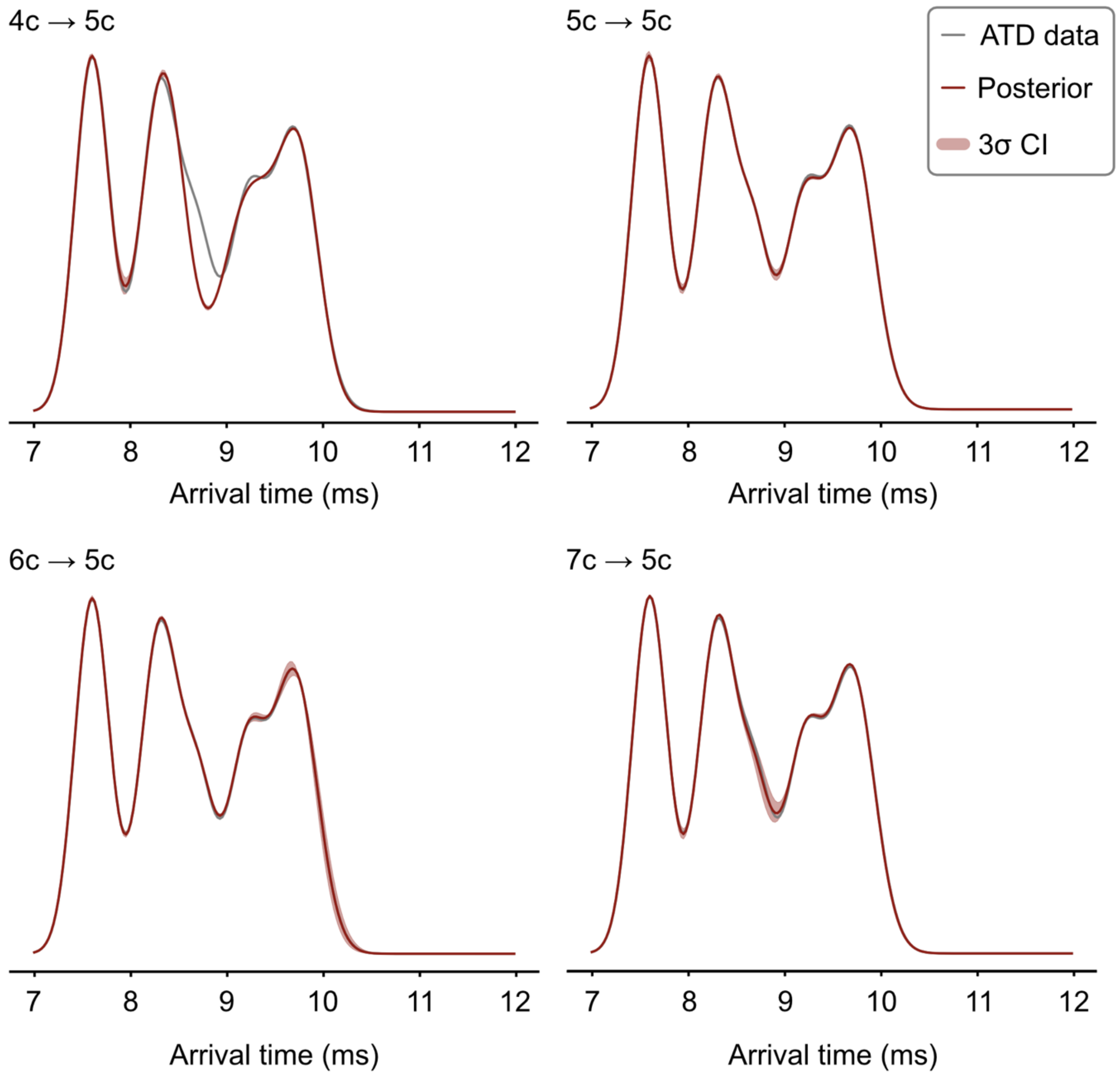

Figure S6. Posterior of the *mc* → 5c deconvolutions after BSP. The 3σ confidence interval and the posterior mean is plotted after drawing 5 x 10^4 random variates from the posterior. The deconvolution 4c → 5c did not converge.

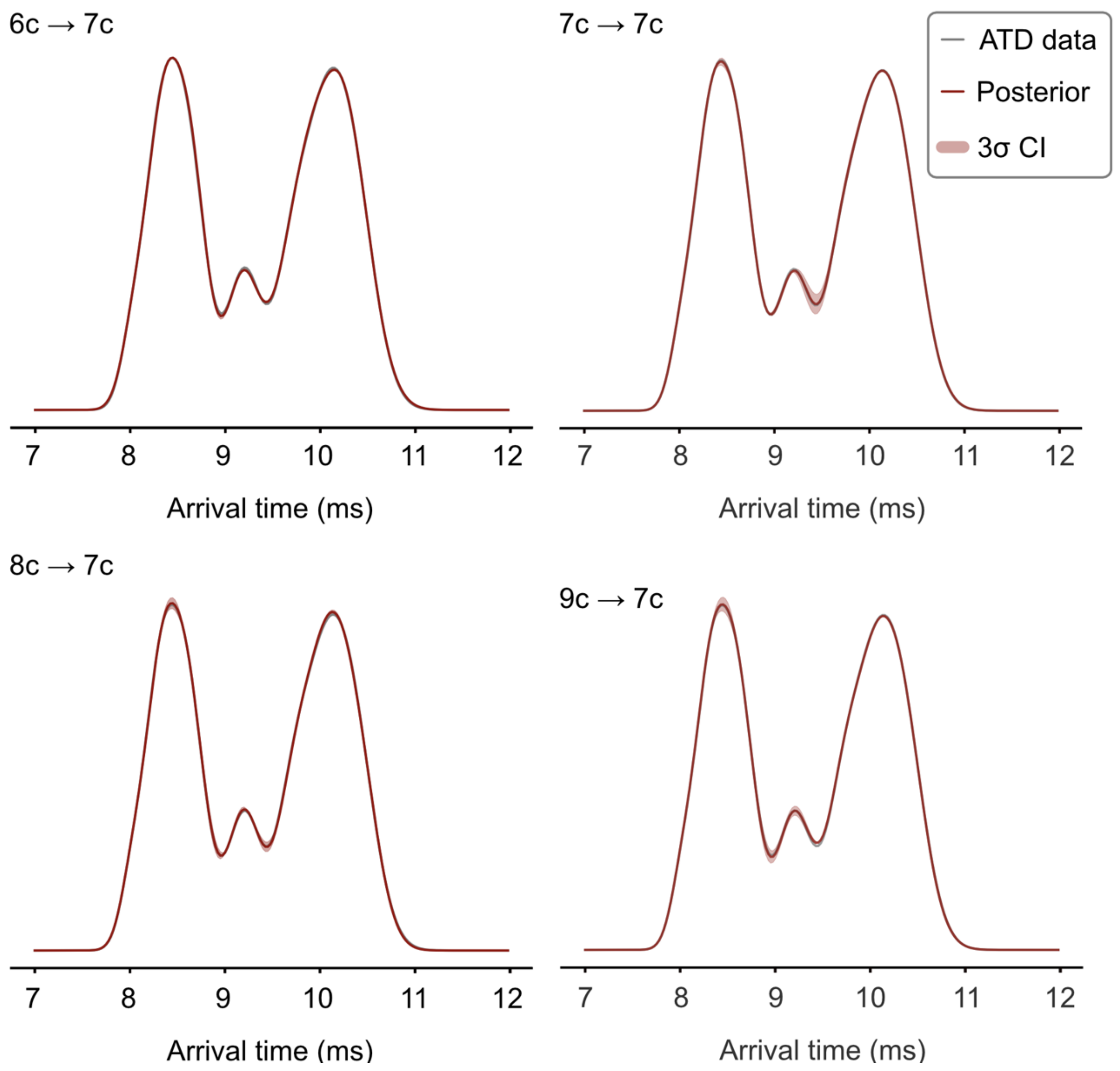

Figure S7. Posterior of the *m*c → 7c deconvolutions after BSP. The 3σ confidence interval and the posterior mean is plotted after drawing 5 x 10^4 random variates from the posterior. All posterior estimates yield a qualitative description of the reference data.

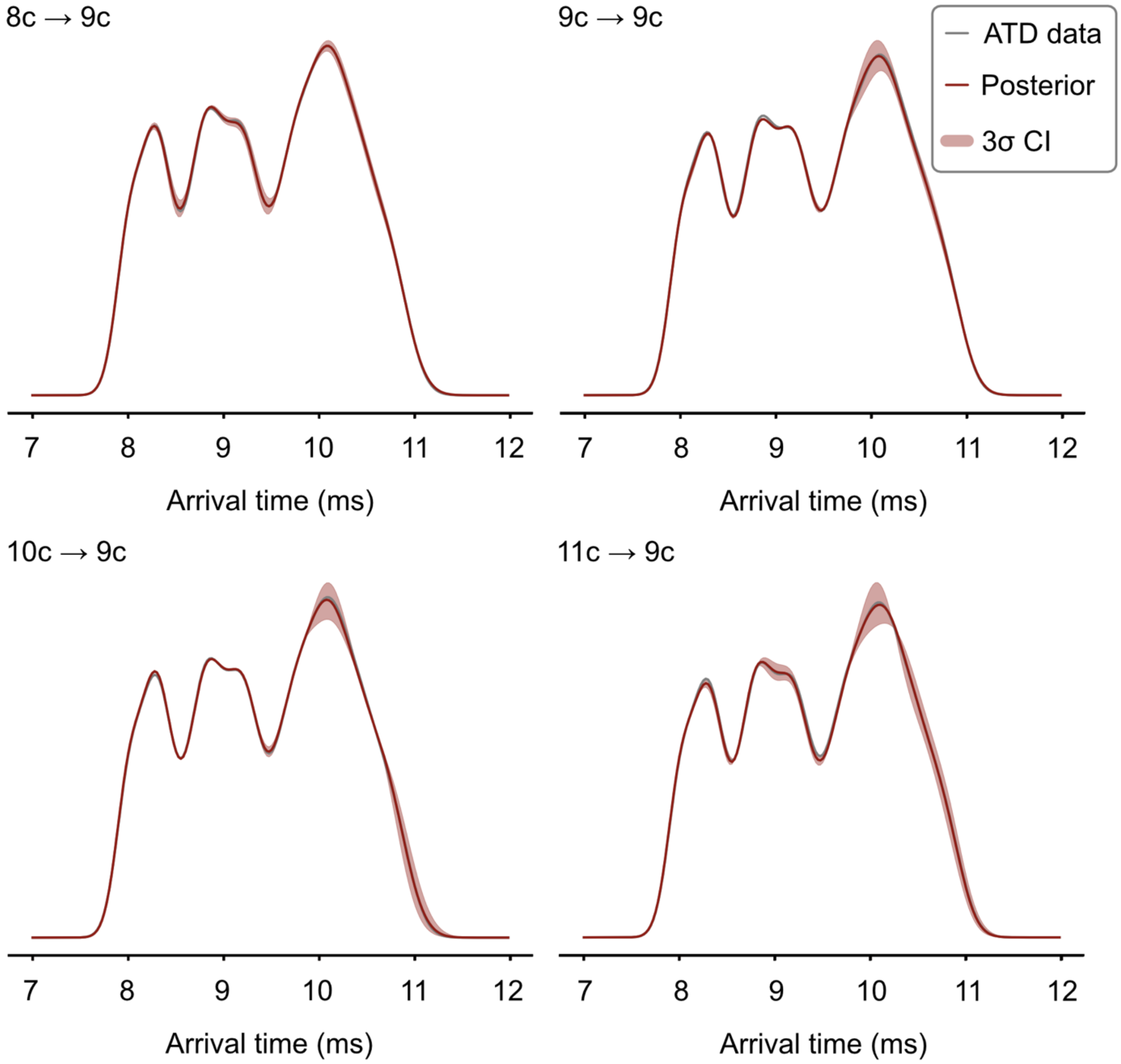

Figure S8. Posterior of the *mc* → 9c deconvolutions after BSP. The 3σ confidence interval and the posterior mean is plotted after drawing 5 x 10^4 random variates from the posterior. In comparison to the *mc* → 5c and *mc* → 7c deconvolutions, the 3σ interval is larger, especially in the region of uniform ion density above 10 ms.

9. Cytochrome c Mass Spectra

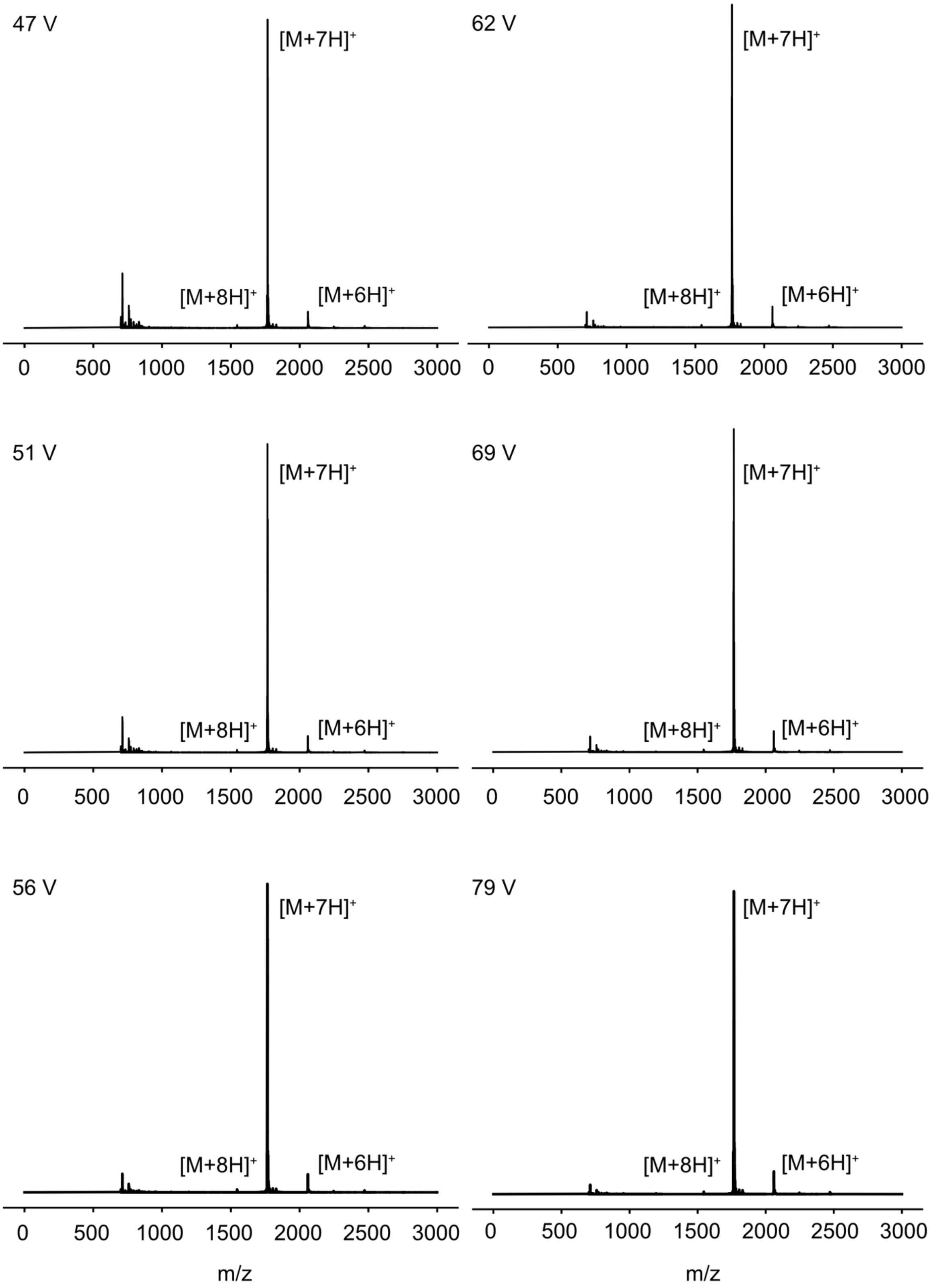

Figure S9. Mass spectra of cytochrome c for every drift voltage in the ATD deconvolution. The mass spectrum is dominated by the solvent-free 7+ charge state (*m/z* = 1766).

10. Deconvolution of Cytochrome c 7+ CCS Data

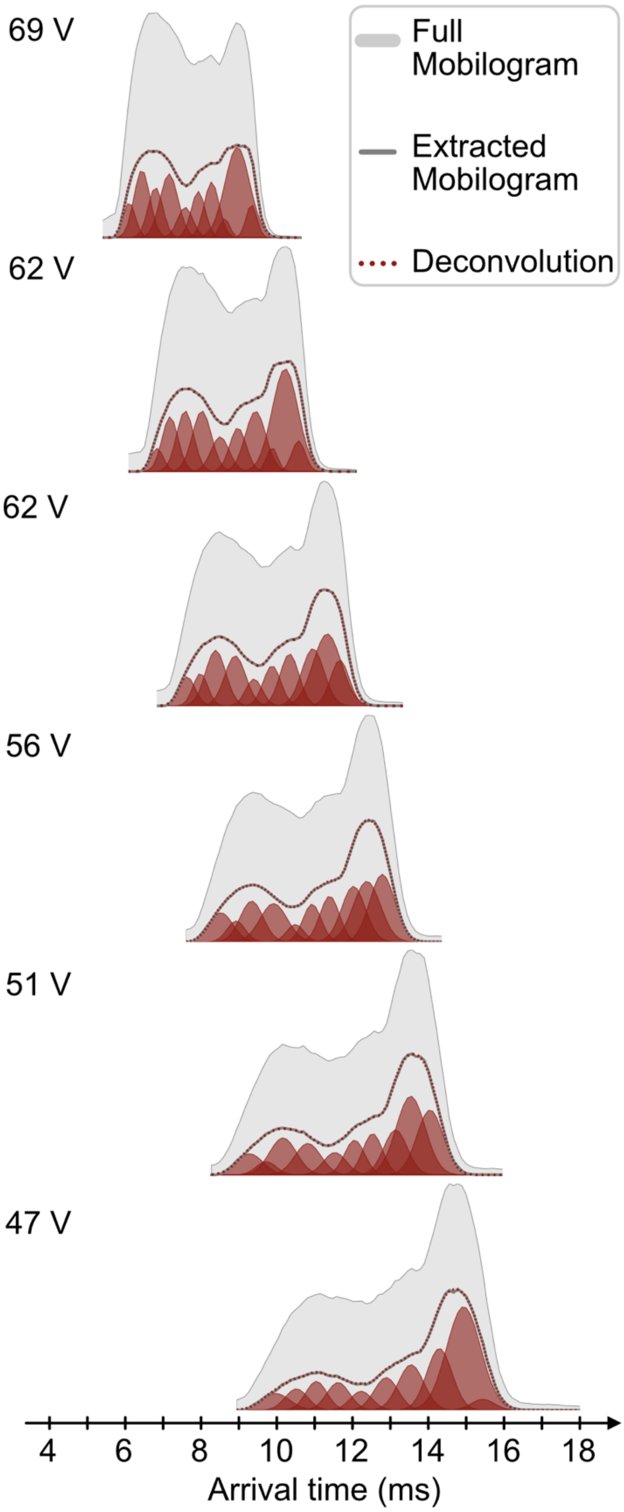

Figure S10. Deconvolution solutions across all drift voltages for the dominant number of components predicted after RJMCMC. The deconvolution solution was obtained by taking the ion background into account for additional ion cloud dispersion.

11. Deconvolution of Cytochrome c 7+ CIU Data

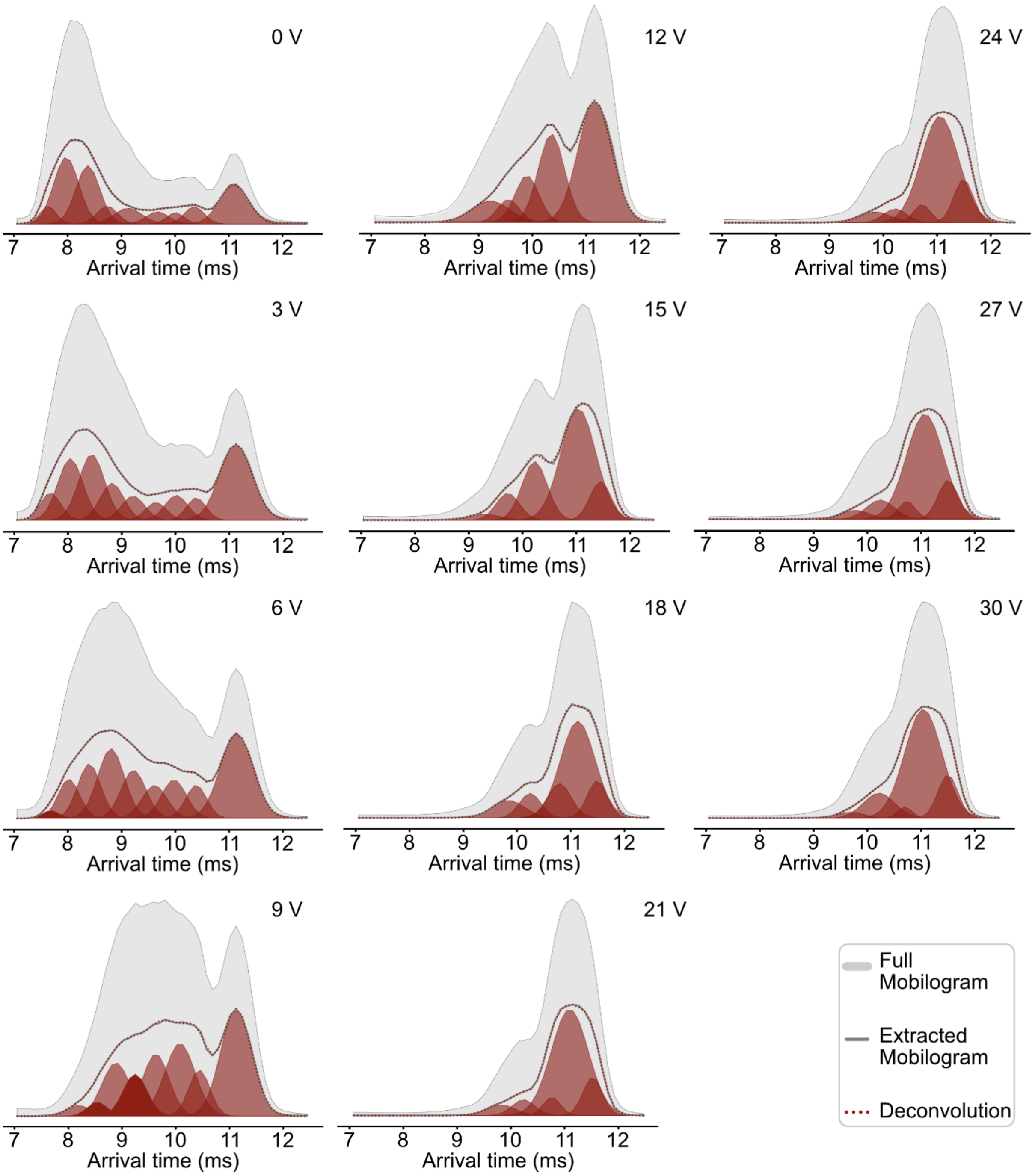

Figure S11. Deconvolution results for the predicted dimensions for every collision voltage probed in the CIU experiment. The deconvolution solution was obtained by taking the ion background into account for additional ion cloud dispersion.

12. PGM *O-Glycan* measurements

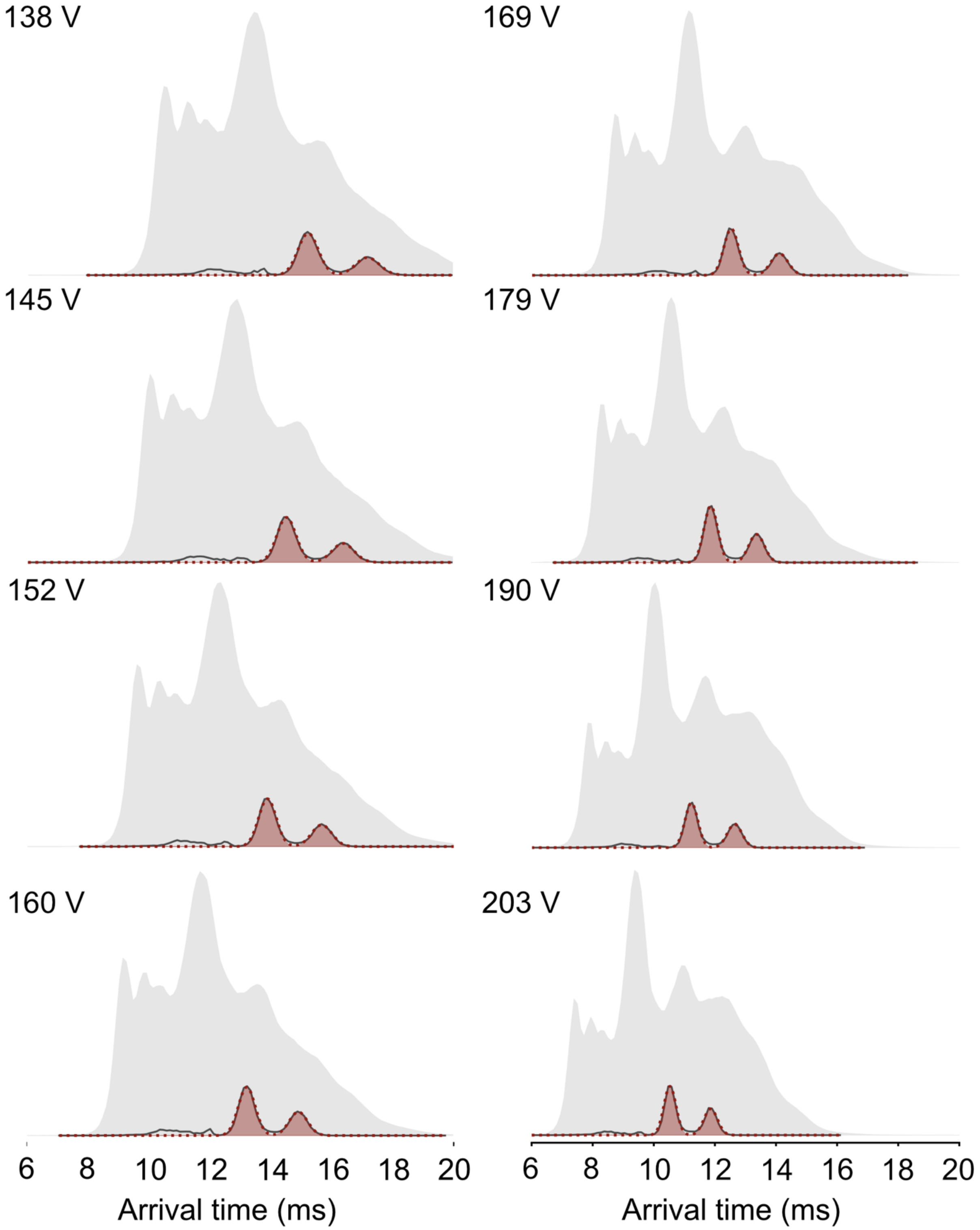

Figure S12. Deconvolution of PGM *O*-glycan with *m/z* 749 across all drift voltages that were used in the calculation of the collision cross section after 10x magnification of the extracted ion count.

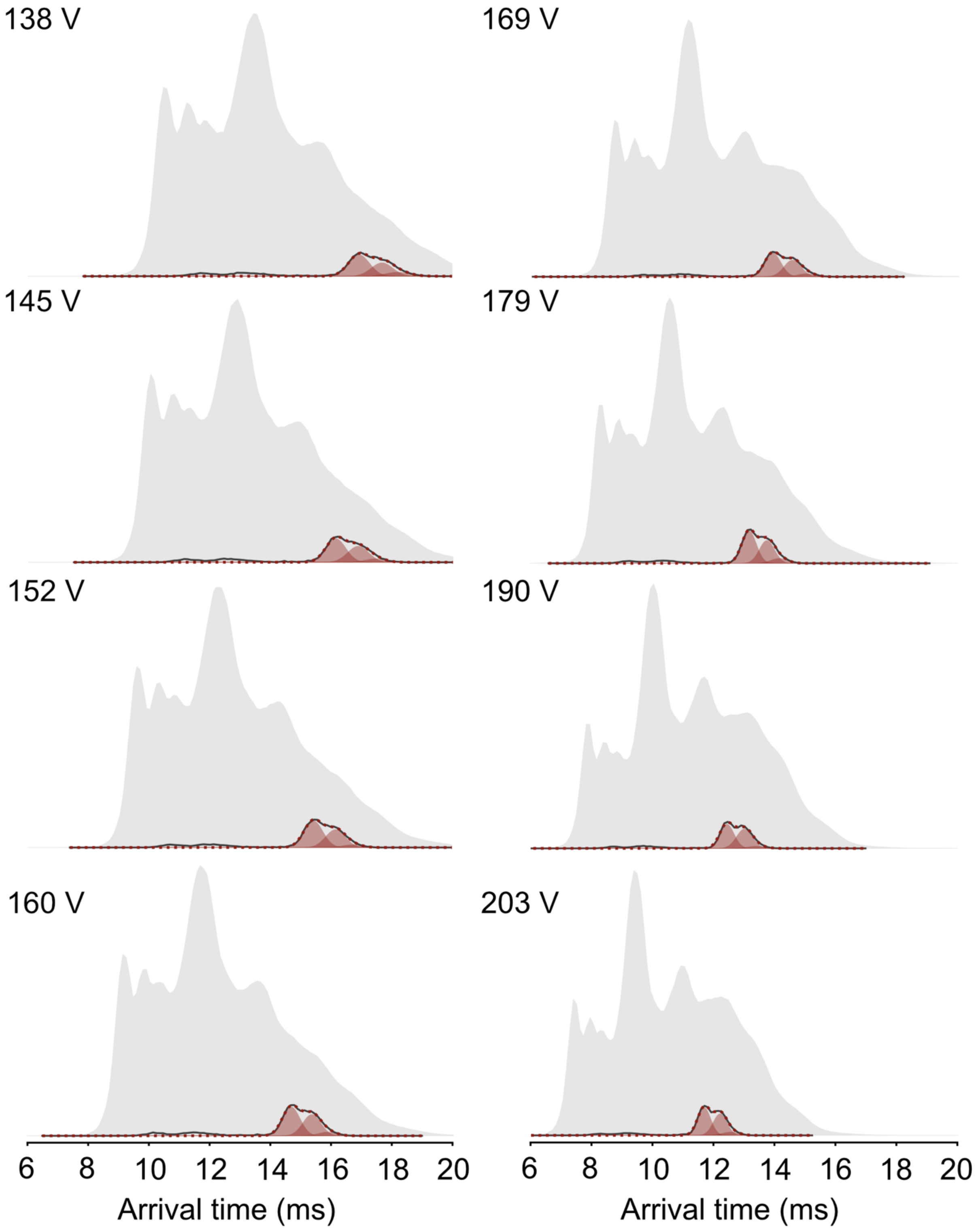

Figure S13. Deconvolution of PGM *O*-glycan with *m/z* 895 across all drift voltages that were used in the calculation of the collision cross section after 10x magnification of the extracted ion count.

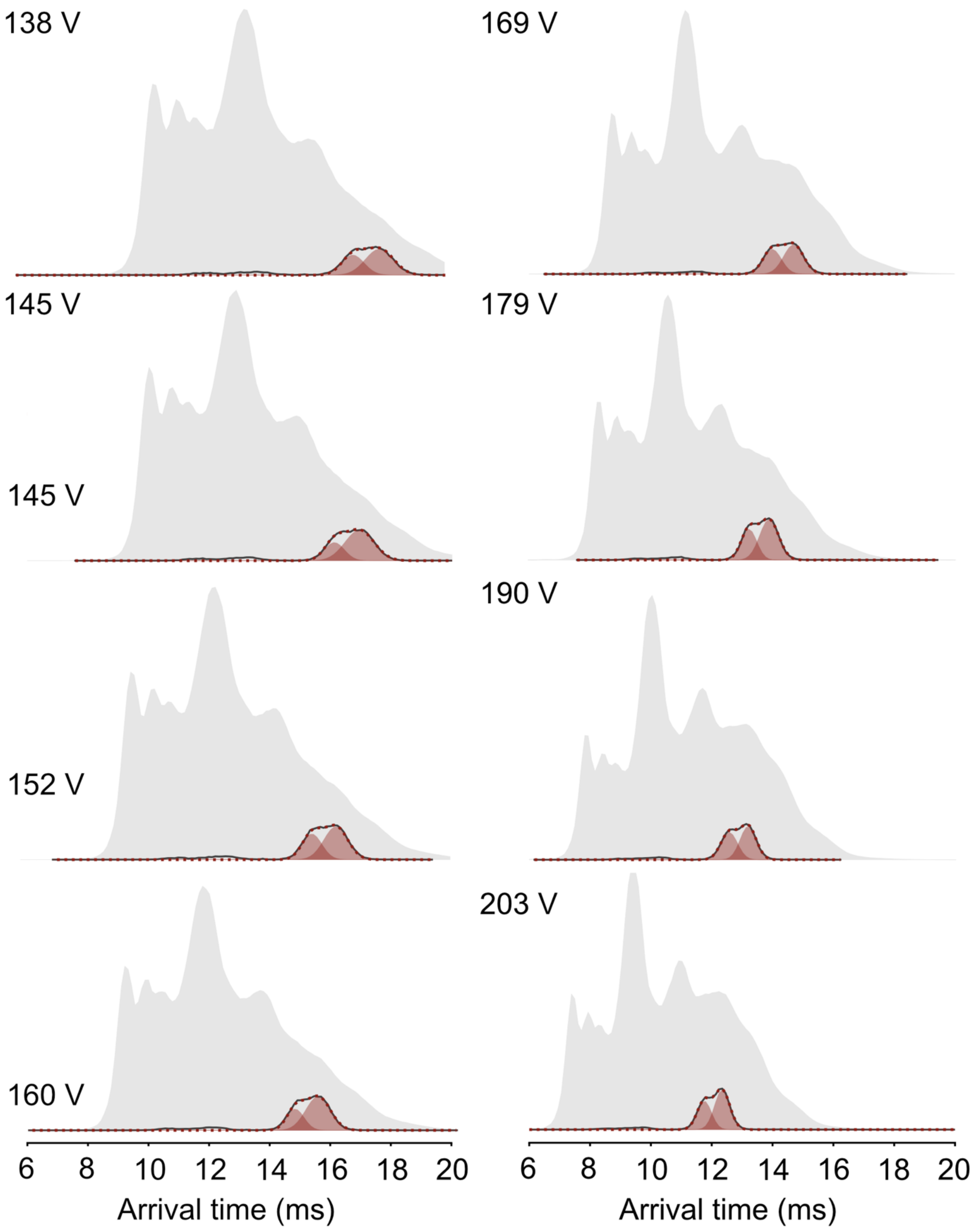

Figure S14. Deconvolution of PGM *O*-glycan with *m/z* 952 across all drift voltages that were used in the calculation of the collision cross section after 10x magnification of the extracted ion count.

13. References

(1) Lindley, D. V. *The Use of Prior Probability Distributions in Statistical Inference and Decisions*; Berkeley, 1961.

(2) Grabarics, M.; Lettow, M.; Kirk, A. T.; von Helden, G.; Causon, T. J.; Pagel, K. Plate-Height Model of Ion Mobility-Mass Spectrometry. *Analyst* **2020**, *145* (19), 6313–6333.

(3) Siems, W. F.; Wu, C.; Tarver, E. E.; Hill, H. H. J.; Larsen, P. R.; McMinn, D. G. Measuring the Resolving Power of Ion Mobility Spectrometers. *Anal Chem* **1994**, *66* (23), 4195–4201.

(4) Tolmachev, A. V; Clowers, B. H.; Belov, M. E.; Smith, R. D. Coulombic Effects in Ion Mobility Spectrometry. *Anal Chem* **2009**, *81* (12), 4778–4787.

(5) Hastie, T.; Friedman, J.; Tibshirani, R. The Elements of Statistical Learning. **2001**. https://doi.org/10.1007/978-0-387-21606-5.

(6) Chib, S.; Greenberg, E. Understanding the Metropolis-Hastings Algorithm. *Am Stat* **1995**, *49* (4), 327–335. https://doi.org/10.1080/00031305.1995.10476177.

(7) Liang, F.; Liu, C.; Carroll, R. *Advanced Markov Chain Monte Carlo Methods: Learning from Past Samples*; Wiley , 2010.

(8) Chib, S. *Markov Chain Monte Carlo Methods: Computation and Inference*; 2001.

(9) Kou, S. C.; Zhou, Q.; Wong, W. H. Equi-Energy Sampler with Applications in Statistical Inference and Statistical Mechanics. *Annals of Statistics*. Institute of Mathematical Statistics 2006, pp 1581–1619. https://doi.org/10.1214/009053606000000515.

(10) Schreck, A.; Fort, G.; Moulines, E. Adaptive Equi-Energy Sampler: Convergence and Illustration. *ACM Transactions on Modeling and Computer Simulation* **2013**, *23* (1). https://doi.org/10.1145/2414416.2414421.

(11) Hukushima, K.; Nemoto, K. Exchange Monte Carlo Method and Application to Spin Glass Simulations. *J. Phys. Soc. Jpn.* **1996**, *65*, 1604–1608. https://doi.org/https://doi.org/10.1143/JPSJ.65.1604.

(12) Shinotsuka, H.; Nagata, K.; Yoshikawa, H.; Mototake, Y. I.; Shouno, H.; Okada, M. Development of Spectral Decomposition Based on Bayesian Information Criterion with Estimation of Confidence Interval. *Sci Technol Adv Mater* **2020**, *21* (1), 402–419. https://doi.org/10.1080/14686996.2020.1773210.

(13) Nagata, K.; Sugita, S.; Okada, M. Bayesian Spectral Deconvolution with the Exchange Monte Carlo Method. *Neural Networks* **2012**, *28*, 82–89. https://doi.org/10.1016/j.neunet.2011.12.001.

(14) Green, P. J. Reversible Jump Markov Chain Monte Carlo Computation and Bayesian Model Determination. *Biometrika* **1995**, *82* (4), 711–732.

(15) Zhang, Z.; Luk Chan, K.; Wu, Y.; Chen, C. *Learning a Multivariate Gaussian Mixture Model with the Reversible Jump MCMC Algorithm*; Kluwer Academic Publishers, 2004; Vol. 14.

(16) Waagepetersen, R.; Sorensen, D. *A Tutorial on Reversible Jump MCMC with a View toward Applications in QTL-Mapping*; 2001; Vol. 69.

(17) Chavis, J. T.; Cochran, A. L.; Earls, C. J. CU-MSDSp: A Flexible Parallelized Reversible Jump Markov Chain Monte Carlo Method. *SoftwareX* **2021**, *14*, 100664. https://doi.org/10.1016/J.SOFTX.2021.100664.

(18) Lu, L.; Jiang, H.; Wong, W. H. Multivariate Density Estimation by Bayesian Sequential Partitioning. *J Am Stat Assoc* **2013**, *108* (504), 1402–1410. https://doi.org/10.1080/01621459.2013.813389.

(19) Majdara, A.; Nooshabadi, S. Nonparametric Density Estimation Using Copula Transform, Bayesian Sequential Partitioning, and Diffusion-Based Kernel Estimator. *IEEE Trans Knowl Data Eng* **2020**, *32* (4), 821–826. https://doi.org/10.1109/TKDE.2019.2930052.
(20) Ng, K. W.; Tian, G.; Tang, M. *Dirichlet and Related Distributions*; Wiley, 2011. https://doi.org/10.1002/9781119995784.
(21) Wegman, E. J. Nonparametric Probability Density Estimation: I. A Summary of Available Methods. *Technometrics* **1972**, *14* (3), 533–546. https://doi.org/10.1080/00401706.1972.10488943.
(22) Krishnamoorthy, K. *Handbook of Statistical Distributions with Applications*; Chapman and Hall/CRC, 2006. https://doi.org/10.1201/9781420011371.
(23) Ondřej Čertík. Symengine: A Symbolic Manipulation Library. Github 2023. https://github.com/symengine/symengine (accessed 2024-01-29).
(24) Allen, S. J.; Giles, K.; Gilbert, T.; Bush, M. F. Ion Mobility Mass Spectrometry of Peptide, Protein, and Protein Complex Ions Using a Radio-Frequency Confining Drift Cell. *Analyst* **2016**, *141* (3), 884–891. https://doi.org/10.1039/C5AN02107C.
